# Abstracts of papers presented at the 1996 Pittsburgh Conference

**DOI:** 10.1155/S1463924696000077

**Published:** 1996


Journal of Automatic Chemistry, Vol. 18, No. 3 (May-June 1996), pp. 71-114

### Abstracts of papers presented at the 1996 Pittsburgh Conference

*The following are the abstracts of the papers read at March 1996s Pittcon which are important to readers of 'Journal of Automatic Chemistry' 1997s Pittsburg Conference will be held in Atlanta, Georgia, from 16-21 March. This will focus on the world of chemistry, instrumentation and innovation. Details from the Pittsburgh Conference, 300 Penn Center Boulevard, Suite 332, Pittsburgh, PA 15235-5503, USA, or follow the information on the internet: http:www.pittcon.org.*

### Development of molecular imprints for residue analysis

*Larry H. Stanker and Mark T. Muldoon, Immunochemistry Research Group, Food Animal Protection Research Laboratory, USDA-ARS, College Station, TX 77845-9594, USA*

Development of molecular imprinted polymers is a technology that utilizes functionalized polymers formed in the presence of a 'print' molecule (the analyte of interest). The polymer is then ground into a fine powder and the analyte extracted from the polymer leaving a molecular imprint of the analyte in the polymer. Molecular recognition is attributed to the formation of functional groups in a particular arrangement within the polymer matrix which conforms to that of the print molecule used in polymer synthesis. Shape-selective cavities also are thought to contribute to analyte binding.

The authors have developed molecularly imprinted polymers to the following agrochemicals: atrazine, a commonly used herbicide; salinomycin, a widely used anticoccidial drug; and ceftiofur, an antibiotic used in veterinary medicine. The use of these MIPs as reagents for the development of analyte specific MIAs has been investigated. In the case of atrazine, specific binding was significantly influenced by the type of solvent used. Organic solvents resulted in increased binding of analyte to the atrazine. The use of MIPs as the solid phase for HPLC has also been investigated; results with the atrazine MIP demonstrated that various *s*-triazines were selectively retained. Capacity factors ranging from 0 to 30 were observed.

The use of MIPs as solid phase matrices for extraction and clean-up of biological sample extracts has been studied. An anti-atrazine polymer was used to clean up organic extracts of beef tissues. In these experiments a small solid phase extraction was prepared containing 500 mg of the polymer. Tissue extracts were applied to the column and contaminants were washed through. Atrazine retained on the column was then eluted from the column by increasing the polarity of the solvent. Both reversed-phase HPLC and immunochemical (ELISA) analyses were performed on column eluent fractions. Following molecularly-imprinted solid phase extraction (MISPE), a recovery of 103% was observed. Analysis of the crude tissue extracts from the 0·1 ppm atrazine-spiked beef liver by ELISA gave a recovery of 9%, however, following MISPE the analyte recovery increased to 57%.

These experiments demonstrate that compounds are present in crude extracts of beef tissue that inhibit the ELISA, but these interfering compounds could be readily removed using MISPE. Thus, MISPE, in combination with an immunoassay such as an ELISA, represents a rapid, inexpensive system for analysis of residues in complex biological matrices and for high sample throughput environmental minitoring programs.

### Analysing foodborne disease: gene probes PCR and epidemiology

*Walter E. Hill, Karen C. Jinneman and Bradley J. Tenge, US Food and Drug Administration, Seafood Products Research Center, PO Box 3012, Bothell, WA 98041, USA*

For the past decade, genetic methods have been used to detect, identify and, to a lesser extent, enumerate micro-organisms in food. The primary techniques employed are gene probe-based DNA hybridization procedures, including colony hybridization and dot blots. More recently, DNA amplification protocols, notably polymerase chain reaction (PCR), have been used to detect and identify foodborne bacteria and viruses. A primary advantage of these genetic methods is that the target organism(s) need not be present in pure culture, thus saving the time required for isolation, purification, and biochemical testing.

During the development of a gene probe or PCR-based testing method, many decisions, such as the selection of appropriate gene targets, the preparation of the sample for analysis, and the optimization of hybridization or amplification conditions, must be made. Thus, while the principles involved in these methods are relatively easy to understand, the application of gene probes and PCR to the testing of foods is often filled with pitfalls and frustrations, such as the presence of PCR-inhibiting substances in food. Other considerations, such as detection method sensitivity and specificity, detection methods, and interpretations of results, need to be dealt with. For example, the level of indigenous microflora plays a role in gene probe sensitivity. Therefore, culturing of the sample and preparation of target DNA are key features and methods often vary from food to food.

Other genetic methods, such as ribotyping, pulse-field gel

0142-0453/96 $12.00 © 1996 Taylor & Francis Ltd.

electrophoresis and DNA sequencing may be used to subtype bacterial strains and thus provide important epidemiological data for the analysis of outbreaks of foodborne disease. The patterns generated by applying these genomic characterization methods are compared by using numerical pattern matching tools. The comparison of patterns yields a matrix of similarity values between 'unknown' test specimens and those of authenticated library specimens. To compare bacterial isolates from food and patient samples obtained during an outbreak, or from fish specimens whose identity is unknown, trees drawn by cluster analysis or the generation of principal co-ordinate analysis graphics are used to visualize the relationships. Pattern analysis is then used to determine the quality of a match reported for an unknown test specimen and the most similar individual or class of individuals (nearest neighbour(s)) in the standard specimen library.

### Automatic nitrogen/protein determination using the dynamic flash combustion method as an alternative to the Kjeldahl method

*Liliana Krotz, Luigi Ragaglia and Franca Andreolini, Fisons Instruments, Strada Rivoltana, 20090 Rodano, Italy and Henry A. Hancock, CE Elantech Inc., 179 Oberlin Avenue North, Lakewood, NJ 08701, USA*

The determination of nitrogen and protein concentration in food, animal feed, fertilizer and related products provides important information for product characterization, nutritional studies and product packaging labelling. The traditional method for nitrogen/protein determination is based on a classical wet chemistry procedure developed by Kjeldahl in 1883. The flash combustion method which was proposed in this paper as an alternative to Kjeldahl method offers simpler, faster and more reliable results without all the inherent problems of the classical method. The flash combustion method does not use toxic or noxious reagents, nor does it require special chemical handling facilities (for example fume hood, special chemical disposal programs etc.).

Dynamic flash combustion, generating high temperatures, instantaneously and quantitatively converts the sample into its elemental combustion gases. These combustion products ($CO_2$, $H_2O$, $N_2$) are swept by an inert carrier gas first through a series of filters that adsorb $CO_2$ and $H_2O$ and then through a gas chromatographic column; finally $N_2$ is introduced to a thermal conductivity detector (TCD). The TCD provides a response which is proportional to the concentration of the sample components, this signal may be acquired and processed to provide a report characterizing the sample component concentration.

Dynamic flash combustion is not matrix dependent and thus can be applied for the analysis of nitrogen/protein in a variety of samples, such as food, animal feed and agriculture products. The solid samples are weighed in tin containers and are automatically introduced into the combustion reactor of the elemental analyser. Liquid samples, such as juice, wine or beer, are introduced by means of a syringe and can be automated using a specially developed autosampler.

This paper presented a thorough evaluation of the flash combustion method using a fully automatic analyser. Comparison of results on nitrogen/protein using the flash combustion method and the classical Kjeldahl method were presented for several samples.

### Direct determination of low PPB levels of chloroform in wine by splitless headspace injection using a multipurpose sampler and GC-MSD

*Andreas Hoffmann, Gerstel GmbH, 45473 Mülheim an der Ruhr, Aktienstraße 232-234 454 73, Germany and Wolf Rudiger Sponholz, Forschungsanstalt Geisenheim, Fachgebiet Mikrobiologie und Biochemie, v. Lade Straße 1, 65366 Geisenheim, Germany*

During sensory quality testing of wine, an off-flavour was occasionally detected. The source of the off-flavour was found to be due to the presence of chloroform at low ppb levels. The routine determination of chloroform was made possible by increasing the detection limits of an HP-MSD, using a syringe-based headpsace system, coupled with a cryogenically cooled PTV injector. This system allows wine to be analysed directly, with no sample pretreatment, and replaces the time consuming extraction/pre-concentration procedure used originally.

Since there are no known natural sources of chloroform in wine production, an investigation was carried out of the various processing steps to determine the reason for chloroform's presence. It was found that bleaching solutions which were used to clean cross flow filters, that contained free chlorine releasing precursors, produced the chloroform.

### Analysis of aroma components in the head space and the nitrogen/protein concentration in beer, wort and malt products for a better assessment of beer quality

*Liliana Krotz, Gianpietro Mapelli, Luigi Ragaglia, Franca Andreolini and Rausto Pigozzo, Fisons Instruments, Strada Rivoltana, 20090 Rodano, Italy*

For the brewery laboratory involved in the research and quality control of beer, wort and malt products, total nitrogen/protein is important analytical information. Currently, most laboratories use the classical Kjeldahl digestion method with all the inherent problems of long analysis time and high cost per analysis. In addition, this wet chemical procedure includes environmental problems of the use of toxic and hazardous chemicals and the resulting waste disposal procedures.

The American Society of Brewery Chemists (ASBC) has approved the dynamic flash combustion method for the analysis of nitrogen/protein concentration. Based on the quantitative combustion and reduction of the sample, followed by GC separation and detection of the molecular nitrogen, the combustion process is safer, more accurate, and provides faster determination of nitrogen/protein thus overcoming all of the problems of the Kjeldahl procedure.

A comparison of the results achieved with a dedicated nitrogen protein analyser based on the flash combustion method, specifically developed to provide the fully automated and unattended analysis of liquid and solid samples was illustrated in this presentation.

For a complete characterization of the quality of beer it is also necessary to analyse its volatile fraction. This fraction includes a wide range of different compounds such as alcohols, esters, ketones, and diketones most of which are generated during the fermentation of raw materials. Because of its simplicity (neither extraction nor distillation are required), speed, and accuracy, a GC analysis of the static head space above the fermentation process is the method of choice.

This paper reported results on the determination of higher alcohols, esters, dimethylsulphide, diacetyl, and pentadione in the static head space carried out simultaneously using three different detectors (FID, NPD, ECD) on a single HS-GC system. A complete aroma profile was achieved within 11 minutes.

The use of two dedicated analysers for the determination of beer volatile components in the static head psace, plus the nitrogen/protein content in beer, wort and malt products provides a complete analytical profile of the fermentation process.

### A combined electrochemical/STM approach for patterning organic thin films

*Richard M. Crooks, Kwok C. Chan, Johathan K. Schoer, Department of Chemistry, Texas A&M University, College Station, TX 77845-3255, USA and Taisun Kim, Department of Chemistry, Hallym University, Chuncheon, Kang-Won Do, 200-702, South Korea*

Self-assembled monolayers (SAMs) of organomercaptans adsorbed onto Au(111) substrates form excellent lithographic resists. The authors used scanning tunneling microscopy (STM) to reproducibly and selectively pattern nanometer-scale features into the SAM resists. These patterns can be selectively metalized using low-temperature chemical vapour deposition (CVD), and they can be used as ultramicroelectrodes. Both applications were discussed in this presentation.

The quality of STM-defined features would be enhanced if the patterning process was better understood, that is, the nature of the interaction between the tip and resist that ultimately leads to patterning. Therefore, the influence of many experimental parameters on pattern quality and resolution was examined. The two most significant findings were as follows. First, a bias threshold, which is independent of the tip current or the duration of exposure, must be exceeded in order to generate a pattern. Second, there must be a significant level of humidity in the ambient to achieve patterning. These results strongly suggest that the resist is removed through a Faradaic electrochemical process, which occurs in the ultrathin-layer cell defined by a physisorbed layer of water confined between the STM tip and the SAM surface.

### Application of fast chromatography to the continuous on-line monitoring of environmental and process streams

*William M. Coleman III, Luis M. Dominguez and Bert M. Gordon, Bowman Gray Technical Center, R. J. Reynolds Tobacco Company, Bldg 611, 950 Reynolds Boulevard, Winston-Salem, NC 27102, USA*

This presentation described a new gas chromatography based analysis system for the measurement of Volatile Organic Compounds (VOCs). Parallel processing of each gaseous sample is a key feature and the system is composed of multiple columns in which separations are optimized for fast chromatography. It also contains the required inert valving for stream selection, which, in conjunction with custom application software, allows for automatic calibration of the system at pre-determined times and for the measurement of individual VOCs successively at multiple sampling locations. All components are contained within a standard cabinet that is suitable for use in a control room environment. The system analyses the sample streams automatically and requires minimal operator intervention.

Fast chromatography was demonstrated with a separation of nine different VOCs and carbon dioxide in 100 seconds in a system which continuously measures two sample streams. A second example showed the separation of 15 VOCs in 160 seconds. For both separations, the accuracy and precision have been estimated at greater than 95%.

The system employs other modules that allow it to be employed for EPA compliance measurements. The system's ruggedness and its use as a process monitor were discussed.

### Process chemical imaging: methodologies and applications

*Hannah R. Morris, John F. Turner II and Patrick J. Treado, Department of Chemistry, University of Pittsburgh, 314 Chevron Science Center, Pittsburgh, PA 15260, USA*

Process chemical imaging involves the integration of digital imaging (machine vision) and spectroscopy and their application to process monitoring. Process imaging has several manifestations. The authors used visible/NIR absorption, Raman scattering or fluorescence emission imaging to visualize and quantitate material chemical heterogeneity. In many cases, chemical imaging analyses can be performed in real time. In these cases, material dynamics can be monitored. With this prospect of obtaining dynamic information, industrial and biological processes can be controlled through appropriate feedback.

Process imaging systems employ tunable imaging filters, including liquid crystal tunable filters (LCTF), in combination with macroscopic or microscopic optics coupled to multichannel detectors. Process imaging is used by the authors to monitor biosystems, and a number of industrially relevant materials. Diverse process examples include fluorescence imaging bioassays in liver assist devices, monitoring of adhesion-promotion processes in impact resistant polymers, and monitoring of semiconductor processing. Process image monitoring system design principles were described, and specific applications were considered.

### Principal component analysis (PCA): scatter diagrams and colour overlays for analysing multivariate image data

*David S. Bright, Microanalysis Research Group, Surface and Microanalysis Science Division, Chem. A114 (Bldg 222), National Institute of Standards and Technology, Gaithersburg, MD 20899, USA*

Data sets with two or more registered images of a sample can be routinely obtained from the electron microprobe as well as other analytical instruments. The data sets from the microprobe, for example, consist of X-ray maps which show the spatial distribution of one element in each map. Several images (maps) are combined to show the locations of chemical phases or regions of interest. Colour overlays and scatter diagrams conveniently show data from two or three images. For more images than three, PCA provides two or three good combined images that show as much of the variation in the data as possible, thus reducing the effective dimensionality of the data from $N$ original images to two or three.

The images obtained from PCA, or 'principal component' images (PC images,) can be related to the original data because of these characteristics: the PC images are particular arithmetic combinations of the original images that can be understood from rotations of the scatter diagrams. A PC image bears resemblance to the expression of a phase or compound stoichiometrically in terms of its constituent elements. The original images can be reconstructed from the PC images. The PC images are ordered, the first showing the most variance, the second showing less variance, and so on, so that the later PC images look largely like noise. The first two or three PC images usually contain the bulk of the 'visual' or 'image' information in the data, and are used for colour overlays and scatter diagrams. The later PC images have mostly noise and very little or no 'image' information and are discarded. The original images can be reconstructed from all of, or a selection of the PC images. If all of the PC images are used, the reconstruction is exact. If the first few PC images are used, the reconstructed images can be inspected to determine if the useful information in the original data has been retained, and thus embodied in those few PC images.

### Chromatography network controller software

*Larry L. Robison, Soheil Saadat and Lisa Fay, Scientific Software, Inc., 3000 Executive Parkway, Suite 530, San Ramon, CA 94583, USA*

The convenience and security of Local Area Networks (LAN) have made their use common in today's laboratories. LANs are often used to provide a central location for the storage of data and method files, as well as to provide common printing resources. Among the limitations of personal computer (PC) based systems for chromatographic use are that the sample sequence batches had to be created and run at the computer workstation attached to the chromatograph and there has been no way to monitor their status from another workstation on the network. This paper described a new network controller designed for use with the EZ Chrom Chromatography Data System.

This EZRemote Network Controller runs on a Windows-based PC equipped ith EZChrom. It enables the user to monitor the status of the other chromatography data systems on the network, as well as edit or create sample sequence batch tables to be used with any of the instruments attached to those data systems. The batches can be downloaded to the appropriate workstation instruments and started or stopped from the remote controller workstation.

The status items to be monitored can be selected from a list and include instrument name, instrument status, detector channels (up to four per chromatograph), run number, sample identification, method, data file name, vial number, run elapsed time. Various configurations can be saved and recalled to suit the needs of the users.

A complete description of the system and examples of its use were presented.

### More LIMS alternatives to traditional LIMS selection and design

*Richard D. Beaty, Vision Technologies, Inc., P.O. Box 556, Pine, CO 80470, USA*

Laboratory Information Management Systems (LIMS) have, since their inception, carried with them a certain mystique, which has set LIMS apart from other software. The image evoked by the word, 'LIMS', actually incorporates much more that just software. This paper dissected LIMS into its component parts of software and associated issues, which together give LIMS its special status *and its special price.* The presentation finished by suggesting an alternative to what has traditionally been incorporated in a LIMS purchase, and evaluated the economy of such an alternative.

A very large portion of the price of commercial LIMS products goes to defray overhead costs incurred by LIMS vendors. Some of the overhead issues include:

(1) *Technical factors:* expensive hardware and operating system requirements; costly and complex underlying database software; extensive requirement for manpower-intensive customization; and extensive requirement for manpower-intensive after-the-sale support.
(2) *Market driven factors:* limited market size over which to amotize development costs; protracted sales cycles and procedures which increase cost-of-sales; and delayed payment practices which add to cost of credit.

This paper examined each of these factors to see how each affects LIMS prices and what, if anything, LIMS purchasers can do to control these factors in a way which pays off in the form of reduced LIMS prices.

Some of the overhead costs borne by LIMS vendors are, in fact, derived from services performed by the vendor for the purchaser. An example is the costs associated with after-the-sale support. Costs passed on for such items should not be begrudged by the purchaser, since these costs are directly related to value received. On the other hand, other overhead costs which are incorporated in LIMS prices are not associated with value received by the LIMS customer, at least in a technical performance sense. An example is the price impact of the large sales and marketing budgets, which full service LIMS vendors are forced to adopt if they are to successfully complete for business.

It is important to note that none of these overhead costs are directly associated with the value of the actual LIMS software. What might the actual cost of LIMS software be if all of the overhead items were stripped out? If this were possible, would such an alternative be viable? This paper discussed the feasibility of reducing LIMS to a commodity and suggested the types of users who would benefit.

### Migration of laboratory data from a proprietary to Oracle database

*Susie Hom and David Wickham, Beckman Instruments, Laboratory Automation Operations, 90 Boroline Road, Allendale, NJ 07401, USA*

As new technology becomes available, Laboratory Information Management Systems (LIMS) have evolved from vendor's proprietary databases to the more popular and supportable relational databases such as Oracle, Sybase, and Ingres. The shift toward standard tools has made those LIMS systems originally implemented with in-house solutions or vendor's software developed with proprietary tools and databases obsolete.

The need to migrate data resident on a proprietary database to the newer LIMS packages is a major consideration when planning a LIMS installation. While it may be possible to maintain both systems concurrently, maintenance costs and support issues become important limiting factors. Since access to the existing laboratory data is mission critical, there is a requirement to migrate the data in a manner that is both auditable and validated.

This paper provided an interesting look into the methodology used for migrating lab data from a proprietary database to an Oracle LIMS system.

### Tools in a software system to improve validation and productively

*John T. Brady and Michael L. Schechter, Shimadzu Scientific Instruments, Inc. 7102 Riverwood Dr., Columbia, MD 21046, USA*

Today's analysts have ever increasing demands on their time. One reason for this is the increased requirements for verification of system performance.

The authors introduced a chromatographic software system that helps meet this challenge. One tool in this system is Smart Batch. Smart Batch allows for the programming of each line in a sequence table with a distinct run type. An action can also be programmed for each line in the table depending on results. One example of run type is system suitability: if the results of the system suitability are in the expected range, the batch will continue; if not, the batch will be aborted. System suitability software can be set with up to 16 separate parameters, plus system RMS noise and drift, allowing for a wide range of acceptance criteria to be set.

Another run type is QC check standard. Five separate levels of QC check standard can be interspersed in the batch. If the programmed criteria are not met, the batch can be halted and a shutdown method can be run preventing precious samples from being wasted.

Other features that help validation are user log and there is a GLP switch preventing the overwriting of file names if a sample has been rerun.

A feature of the instrumentation is the ability to program sample dilutions and reagent additions in the Smart Batch table. Autoconfiguration provides positive instrument component identification in reports.

Auto-Launch increase productivity allows for a computer to go directly into the CLASS-VP instrument module designated in the CLASS-VP ini file. Remote start gives the ability to start and monitor a batch in the laboratory from another location.

### LIMS and chromatographic data acquisition in the manufacturing environment

*Mary D. Hinton and Phillip R. Hinton, Applied Computer Systems, 3540 Country Ct. N., Mobile, AL 36619, USA*

Chromatographic analyses often make up the majority of the tests performed in pharmaceutical, petroleum, and chemical plant laboratories. In order to fully automate a laboratory, the LIMS must work closely with the chromatographic data acquistion system. Typical functional requirements that will facilitate a smooth coupling of the two systems were presented for both the data acquisition system and the LIMS. A detailed discussion on how these two systems should work together to manage the information flow in a manufacturing plant was also given.

It is expected that the newer data acquistion systems should have some built-in data management capabilities. The following are data manipulation functions that can be built directly into a chromatographic data acquistion system (these functions could then be integrated with a LIMS for better data management):

(1) Maintain a database of chromatographic data.
(2) Allow each injection to be viewed in real-time.
(3) Allow automatic or manual recalibration.
(4) Allow all the injections in a current run to be viewed simultaneously as each injection is acquired.
(5) Allow the viewing of previously stored runs.
(6) Allow on-the-fly peak identification of unassigned peaks.
(7) Allow data to be re-analysed, when necessary.
(8) If the LIMS cannot access the chromatographic database, use standard file formats (for example include: Andi) for exporting the data.

Functions needed in the LIMS:

(a) Import the data from the data acquisition system into a usable LIMS format. Set up data entry forms to automatically import the peak information from the data acquisition system and perform calculations on the results.
(b) Allow the import of standard results previously stored in the LIMS database to perform calculations.
(c) Maintain material specifications for chromatographic results and determine if results pass material specifications.
(d) Automatically generate a COA (certificate of analysis) with the data results required by the customer specifications.

To isolate the data acquisition system from any problems on the network, a local database can be maintained at the workstation. However, process control needs to be able to obtain the chromatographic results as quickly as possible. When the information is imported to the LIMS, process and other LIMS users will have access to the data without disturbing the technicians' workstation.

### Software design for data acquistion, control and training in a unified instrument: environmental applications of mercury analysis, flow injection, and ion chromatography

*S. Stieg, Lachat Instruments, 6645 West Mill Road, Milwaukee, WI 53218-1239, USA*

With the advent of performance-based method regulation for USEPA compliance monitoring, the regulated laboratory itself will need to become more flexible and to choose methods and techniques best suited to the tasks at hand, and not simply because they are 'approved' methods. Special-purpose instruments, labs and personnel training, designed for only one determination per sample and only one technique, can be the limiting step in a commercial testing or environmental monitoring laboratory.

A commercially-available instrument design platform, the Lachat instruments 'QuikChem Continuum' series and its software, the 'Omnion Continuum' platform, attempts to create a unified approach to instrument flexibility. By addressing the common aspects of a mercury cold vapour analyser, multichannel flow injection analysis (FIA), and ion chromatography (IC), much of these several techniques hardware and software can be combined, reducing the support cost of the several techniques, and encouraging cross-training and generalism in the training and development of lab personnel. A generalist approach to training encourages thinking and empowerment which can reduce turnover.

The heart of the Omnion Continuum is the Shared Peripheral System (SPS). The SPS presently consists of two Windows 3.1 applications, 'autosamp.exe' and 'pumpctri.exe', These two applications are launched by any of the Omnion programs, at log-in time, and are then available to take on from one to eight 'instrument clients'.

The autosamp.exe application manages requests from the instrument clients for shared autosampler and dilutor service. For example, if an ion chromatography instrument (first client) needs a sample with a 10-fold dilution at the same time that a three-channel FIA instrument (second client) needs a different sample, the first instrument client to make the request will receive the full attention of the autosampler and dilutor. The second client will be placed in a queue, for service when the autosampler and dilutor is finished with the first client's request. If more than one autosampler and dilutor are available, the two clients can be served in parallel, with no waiting in a queue necessary.

Since all three types of instruments generate one or several peaks per sample, it is easy to plan a unified approach to control data acquisition, and information export. Examples of IC (several peaks per sample), FIA (one peak per sample) and mercury cold vapour atomic fluorescence (one peak per sample) data acquired by the unified Omnion system were given.

### Multiple databases for laboratory information reporting and storage

*Gary F. Gostecnik and Michael A. Epton, Analytical Automation Specialists, Inc., 11723 Sunbelt Court, Baton Rouge, LA 70809, USA*

Prior to computerization, laboratory data were recorded notebooks and copies of reports were stored in file cabinets after distribution. Early computerized Laboratory Information Management Systems (LIMS) followed this same concept of retaining data in the laboratory. Often the long-term costs of maintaining and retrieving this computerized information were underestimated. With the reductions and restructuring of the past five years, many laboratories found a disproportionately large share of their budget going to maintain the LIMS.

Once released by the laboratory, the data logically should become the property of the party requesting the analysis. Currently, distributed systems such as PC networks are increasingly being employed for facilitating the reporting and maintaining of laboratory information. Most departments requesting lab data employ electronic tools such as spread-sheets and databases, The use of Open Database Connectivity (ODBC) to automatically update databases outside of the laboratory after the data have been released, enhances the movement of data for utilization by the requester and eventual storage. Cost reductions are realized through less laboratory involvement in these functions. Additionally, the concurrent increase in performance due to the reduction in laboratory system overhead provides an added incentive for the use of multiple databases.

### Integrated information management: strategy and practice

*Andrew J. Grygiel, Dan Holmes and Michael Rank, Hewlett Packard Company, 1601 California Avenue Palo Alto, CA 94304, USA*

The focus for business process improvement in the laboratory has been shifting rapidly in the past few years. Today, the greatest opportunities for enhancing laboratory productivity lie in the areas on either side of the traditional laboratory instrument: sample preparation and information management. The latter area, information management, can provide the greatest potential for increased laboratory efficiency if performed in an integrated, comprehensive, and user-friendly fashion. An Integrated Information Management (IIM) environment contains standards-based, scaleable, computing solutions that grant laboratory personnel access to the information that they want, where they want it and when they want it.

This paper described a strategic framework for developing Integrated Information Management (IIM) solutions, and the practical steps by which a laboratory can achieve the IIM vision. How IIM was obtained, and the enterprise-wide benefits, and the enabling technologies that make realization of an IIM laboratory possible were described.

This work illustrated key aspects of IIM through real-world examples, drawn from a broad spectrum of laboratories representing the pharmaceutical, chemical, and environmental industries.

### Use of Rapid Application Development (RAD) programming techniques in the development of complete analytical solutions

*Scott J. Hein and Terry L. Ramus, Diablo Analytical, Inc., P.O. Box 5889, Concord, CA 94524, USA*

One of the recent trends in the development of analytical instrumentation is the emphasis on producing complete analytical solutions rather than just analytical hardware. A major challenge associated with providing a complete solution is to address the unique needs of each customer through some level of customization. Frequently the custom content of a solution is related to special data acquisition or data analysis requirements of the user's operating environment. These special requirements can often be addressed through custom software modifications and enhancements to the instrument data system.

Rapid Application Development (RAD) programming techniques are currently used to produce mainstream business applications. RAD programming can also be used to provide the custom content for analytical applications. This presentation described RAD programming techniques and tools, with an emphasis on integration with commercial analytical data systems. Several examples of this approach were presented, including applications in environmental data analysis, industrial quality assurance, and instrument validation.

### User programs for a chromatography data system

*Michael L. Schechter and John Brady, Shimadzu Scientific Instruments, Inc., 7102 River Wood Drive, Columbia, MD 21046, USA*

Today's chromatographer is confronted by the ever-increasing amounts of data produced by their automated data systems. Autosamplers and automated sample preparation systems have markedly increased the throughput of the average laboratory chromatogaph. Without some means of managing this flood of data, all this automation will only increase the production of raw data, not results and productivity.

To increase productivity, Shimadzu CLASS-VP can perform custom calculations. Shimadzu CLASS-VP supports two types of custom Windows application interactions (Custom Parameter and User Program), as well as Dynamic Data Exchange (DDE), and several ASCII data export and import modes. These features allow the chromatographer to increase result productivity by customizing the software to perform many time-consuming applications, and can be used to generate validation results as well.

Shimadzu CLASS-VP Custom Parameter applications perform user-defined custom calculations, which are processed as if they were built into the software. User programs can be called by Shimadzu CLASS-VP either before a run or after analysis. These user programs can access the text files generated by the ASCII export functions. The export files contain the chromatographic results from each integration, and the data can be stored in a number of formats. DDE provides a link to any Windows DDE client, updating the links with new infomation after each analysis. The ASCII import capability allows the user to transfer data from a gel scanner or similar instrument and process the results with Shimadzu CLASS-VP.

This paper described the use of these applications to increase productivity in the laboratory. These applications allow the user to add custom functionality to Shimadzu CLASS-VP, tailoring the software to fit a specific laboratory situation. Examples of custom application programs were discussed.

### Migration from a successful first generation LIMS to a general purpose LIMS system

*Tom P. Oberstar, KRMS-NATLSCO, 95 Oakwood Road, Lake Zurich, IL 60047, USA; Jacqueline Elliott, KNIC, Route 22 & Kemper Drive, Long Grove, IL 60049, USA and Kirk Shelvay, Hewlett Packard Company, 1400 Morris Drive, Wayne, PA 19087, USA*

In 1992, KRMS-NATLSCO laboratory, a consulting laboratory specializing in industrial hygiene analysis, embarked upon the task of identifying a commercially available LIMS to replace and expand upon the capabilities of the in-house developed system. The in-house system was developed in the early 1980s using existing computer hardware and was tailored specifically to meet the needs of the laboratory. The business decision to develop the system internally was based on the lack of a commercially available LIMS which met the laboratory's requirements. Since that time, the LIMS market has matured significantly and, as a result, it was once again attempted to identify a commercially available LIMS which would meet the requirements.

Following the development of a lengthy and detailed requirements document and investigation of a large number of vendors, Hewlett Packard's ChemLMS was the best fit for the authors' laboratory. The rapidly changing requirements of the industrial hygiene laboratory market required that the base system be flexible, scalable, and capable of meeting the most stringent GALP requirements. ChemLMS was chosen primarily because it affords the user the opportunity to develop a system which closely mirrors the operation of the laboratory, rather than the laboratory altering its operations to conform to the structure of the LIMS. In addition, ChemLMS met the objectives of flexibility, scalability, and compliance with GALP requirements and gave the laboratory the ability to leverage ORACLE technology into the new LIMS.

The objectives for KRMS-NATLSCO laboratory in construction of the new LIMS were:

(1) Closer compliance to the intent of GALP.
(2) Total integration of all aspects of laboratory operation.
(3) Maintain the strengths of the original LIMS in the design and implementation of the ChemLMS system.
(4) Allow for greater flexibility and adaptability to changing regulatory and business requirements.
(5) Seamless and transparent integration of external ORACLE tables and technology into the ChemLMS system.
(6) Increased efficiency of operation.
(7) Improved data integrity.
(8) Improved reporting and electronic downloading facilities to meet client demands.

The mission was to meld the features of our existing LIMS into the new system and expand upon its capabilities. This has been the major determing factor in the development process of the new LIMS. Unlike most in-house LIMS developments, the KRMS-NATLSCO LIMS was extremely successful and met the laboratory's needs for the past 10 years. The new LIMS builds on the foundation of the authors' original system offering tighter integration of all aspects of business operation, from supply ordering and sample analysis to reporting, invoicing, and integration with corporate business systems. This paper chronicled the implementation of a ChemLMS LIMS system, the challenges associated with managing the efforts of internal, consultant, and vendor programming resources. This work also described the synergy necessary between LIMS vendor and end user for a LIMS implementation to be successful.

### Calculated GC/FTIR calibration curves for the analysis of reformulated gasolines

*John W. Diehl and Frank P. Disanzo, Mobil Oil Corporation, Paulsboro Technical Center, Paulsboro, NJ 08066, USA*

Federal regulations will soon mandate that the levels of a number of compounds present in reformulated gasolines be monitored and controlled. These include oxygen-containing compounds, such as ethanol and methyl-t-butylether, and aromatic hydrocarbons, such as benzene and naphthalene. The use of GC/FTIR to perform this analysis requires developing internal standard calibration curves for 12 ethers and alcohols (oxygenates) and 26 aromatic hydrocarbons and consequently preparing and chromatographing 25 gravimetric solutions. This paper presented a method for calculating the calibration curve of an analyte from the gravimetrically derived curve of a similar compound which greatly simplified and streamlined the entire calibration procedure. The curves for oxygenates such as ethyl-t-butylether and ethanol were calculated from the slope and intercept of methyl-t-butylether's (MTBE) curve and the curves for aromatics such as 1,3,5-trimethylbenzene and naphthalene were calculated from that of toluene. These calculated curves had an average percentage accuracy of 1·9% which compared favourably with the average percentage accuracy of 0·9% obtained with curves developed from gravimetric standards.

Calibration factors for 38 compounds, data from tests with several spectrometers, and the analyses of fuels with very different properties such as reformates, fluidized catalytic cracking, and blended gasolines were presented.

### Using windows DDE to customize FTIR software behaviour

*Bruce McIntosh and Mitch Mallough, KVB/Analect, 9420 Jeronimo Road, Irvine, CA 92718, USA*

Over the years FTIR vendors have each built proprietary software environments for their instruments. While all get the job done in terms of collecting and processing spectra, each bears the effects of the skills and biases of the software engineers who wrote the packages and, lacking a standardized approach, each package operates differently, creating confusion among users and adding training cost. In addition, no package is likely to behave exactly the way a particular user would want. Galactic Industries and some other companies provide a common interface and programmability in their third party packages, allowing users to define instrument behaviour. However, to take full advantage of this customizability, the user needs to learn a relatively complex programming language.

The wide use of Microsoft Windows has opened up another approach to tackle this customization. The DDE (Direct Data Exchange) protocol allows simultaneously running Windows applications to share data and control one another. Establishing DDE links between DDE aware Windows applications is generally quite easy, employing simple ASCII commands and responses. This presentation discussed this functionality in depth.

### Multichannel data acquisition using a DSP controlled step-scanning infra-red spectrometer

*Dirk D. Laukien and Thomas J. Tague Jr., Bruker Instruments, Inc., Manning Park, Billerica, MA 0182, USA*

In the past, multichannel acquisition of step-scan data has had to use lock-in-amplifiers (LIA). The experimental set-up for these experiments has been daunting to the scientist or technician with little or no experience with electronics. The four most common types of modulation investigations that have been shown to benefit from step-scan acquisition are vibrational circular dichroism, polymer stretching analysis, liquid crystal modulations, and multichannel photoacoustic spectroscopic (PAS) depth profiling. For all of these experiments the scanning mirror is typically modulated at each retardation position, where the demodulation is performed with an LIA. A second LIA is necessary for demodulating a second PAS channel a polymer stretching frequency, or a liquid crystal electric field modulation. The digital signal processing (DSP) mechanism utilized by the Broker series of step-scanning spectrometers allows the above mentioned experiments to be performed without the need of a lock-in-amplifier. The demodulations are performed in Fourier space using a digital processor. The nature of the experimental setups and typical results were presented and compared to those obtained using non-DSP technology.

### Validating the ordinate performance of FTIR spectrometers

*C. M. Deeley and R. A. Spragg, Perkin-Elmer Ltd, Post Office Lane, Beaconsfield, Bucks HP9 1QA, UK*

Recently a list of 50 potential sources of ordinate error in FTIR spectrometers was published. Unfortunately no procedures for establishing that an FTIR spectrometer is producing reliable transmission or absorbance values have been widely adopted. At present, the choice of traceable standards is limited to a glass with certified transmission, or organic liquids with known band intensities. The metits of these were compared.

In practice, it is more difficult to measure transmittance accurately than it is to measure absorbance relative to a baseline. As most analytical measurements use absorbance it would appear best to base routine accuracy tests on this. However, the greatest interest in ordinate accuracy has been displayed by the optical industry where the requirement is to determine transmittance. In that industry there remains considerable scepticism about the accuracy of FTIR spectrometers.

The certified glass sample is robust and convenient. It has an atypical spectrum consisting of broad maxima and minima between about 70% at 400 $cm^{-1}$ and 0% at 2000 $cm^{-1}$. The results are therefore rather insensitive to small differences in resolution and line shape. While this restricts the utility of the material, it does give laboratories a good chance of matching the certified values.

Because the organic liquids have to be contained in relatively short pathlength cells with alkali halide windows, they require a rather elaborate calibration procedure and are inconvenient for frequent use. For most users it is more important to establish that an instrument is giving consistent results than to check absolute accuracy. Therefore the spectrum of a standard should be sensitive to the sort of changes that might occur in an individual instrument as well as to the differences between instruments. The effects of both kinds of variation, within and between instruments, were considered.

### Automatic system diagnostics of a process FTIR instrument using PCR analysis of an on board reference function

*David Marrow, KVB/Analect, 9420 Jeronimo Road, Irvine, CA 92718, USA*

The control and operation of the moving element of an interferometer is the most sensitive and error prone part of an FTIR instrument. Diagnostics of problems, generally occur only after failure and require an expert technical person. In an on-line process application where the instrument is expected to operate uninterrupted for extended periods of time, it is not desirable to trigger service based on a failure. Frequent service to prevent this type of occurrence is also undesirable because of the expense and requirement to take the instrument off line. Ideally the instrument would warn the operator of an impending failure well enough in advance so that a service call could be conveniently scheduled. Work has been done using an FTIR instruments spectrum analysis characteristics to process a electrical reference function injected into the ADC in place of the detector signal. The processed function or spectrum was then analysed using PCA techniques available as part of the system software to develop an indicator function. The result of the analysis were compared to standard instrument performance characteristics and relationships were found between a number such that the output may be used to indicate impending failure. The results of this work were presented.

### Ergonomics: improving laboratory safety and efficiency

*Andrew J. Marcotte, The Joyce Institute/A Unit of Arthur D. Little, Inc., 1313 Plaza 600 Building, Seattle, WA 98101, USA*

Ergonomics, the science that addresses employee performance and well-being, has received considerable attention in the manufacturing sector. In many cases, problem-solving efforts have successfully attacked the increasing rates of musculoskeletal injuries and illnesses such as tendonitis, carpal tunnel syndrome, and back and shoulder strains. The result of workplace changes have also resulted in productivity and quality improvements.

Laboratory environments are very different from manufacturing environments. However, the same types of problems (increased injuries, absenteeism, accidents and errors) are occurring in laboratories. The source of these problems arises from prolonged or repeated use of poorly designed equipment, handling heavy containers, instruments that place excessive physical demands on a small part of the hand, and overall laboratory designs that have not been updated to reflect the changing demands on technicans and employees.

Ergonomic design principles can be applied in the short term to create effective practical solutions to problems in current work procedures, workstation designs, and laboratory layouts. The lessons learned have also been used to establish purchase criteria for test equipment, carts, and instruments. The discussion used case studies to illustrate techniques that have been used both in the short and long term to achieve measurable improvements in safety and area performance.

### Flow injection analysis of aqueous samples for magnesium with a magnesium ion-selective electrode as detector

*Nikolas A. Chaniotakis, John K. Tsagatakis, Department of Chemistry, University of Crete, Iraklion 71409, Crete, Greece, and Steven J. West, Orion Research, Inc., 529 Main Street, Boston, MA 02129, USA*

Magnesium is one of the elements that is routinely monitored in drinking water reservoirs, as well as other aqueous samples, pharmaceutical preparations, and various consumer products. The use of a Flow Injection Analysis (FIA) technique for automated, routine analysis based on potentiometric magnesium Ion-Selective Electrode (ISE) detection is now possible due to recent progress in the development of these sensors.

In this presentation, it was shown that the synergism between the new potentiometric magnesium detector, a pulseless pumping system, and a newly designed flow-through cell have allowed for the simple, direct, and precise determination of the magnesium concentration in water samples

The FIA analyses were performed on a custom-made system with several convenient design features. The flow cell has very low dead volume, approximately 15 ul and the ISE sensing module can be exchanged very easily with virtually no system downtime. The effect of the buffer concentration, reaction time, and electrode pre-treatment were presented in detail. Finally, the use of preloaded cartridges and a syringe pump allow for easy change of reagents which are driven through the system generating negligible streaming potential error at the sensor surface.

The results obtained with the above system compare favourably with those obtained using a flame atomic absorption instrument and at a fraction of the cost.

### Ambient air analysis using high speed gas chromatography

*Anita J. Gorsuch and Mark A. Klemp, Chromatofast, Inc., 912 N. Main Street, Suite 14, Ann Arbor, MI 48104, USA*

An instrument has been developed for the analysis of volatile organic compounds in ambient air using cryo-focusing inlet technology originally applied to liquid analyses. The use of a vacuum pump to pull sample onto the cold trap allows for sampling directly from ambient air or sample containers. By varying the length of time sample is pulled to the trap, an adjustable amount of preconcentration or cryointegration of the sample is possible. This can result in sensitivities in the sub-ppb range when used with flame ionization detectors. In addition, the inlet system does not contain valves in the sample flow path which prevents any sample loss or contamination. Pretreatment of humidified samples is not required since the inlet is highly tolerant to large amounts of water. Techniques for interfacing this technology to either atmospheric or vacuum outlet detectors was shown. These technologies have been integrated with Varian gas chromatographs. Design features and performance characteristics of the sampling system for a variety of compounds were presented.

### Analysis of VOCs in soils by automated static headspace/GC (EPA Method 5021)

*James D. Stuart, Michael E. Miller, Department of Chemistry, U-60, University of Connecticut, Storrs, CT 06269-3060, USA, Valerie Naughton, Denise J. Sherman and Joseph Halloran, Tekmar Co., 7143 East Kemper Rd, Cincinnati, OH 45249, USA*

This study has examined certain of the parameters of EPA Method 5021 found to be important for obtaining accurate and reproducible concentrations of volatile organic compounds (VOCs) from different soils using the automated static headspace GC method. The equipment employs a Tekmar 7000/7050 Headspace Autosampler coupled to a Model 5890 Hewlett-Packard gas chromatograph equipped with a capillary column and flame ionization detector. The target analytes included: benzene (B), toluene (T), ethylbenzene (E) and various xylenes (X) (collectively referred to as BTEX), methyl-*t*-butyl ether (MTBE), ethyl-*t*-butyl ether (ETBE), trichloroethane, trichloroethylene and tetrachloroethylene. Generally, the relative recoveries of the BTEX, the two oxygenates and the chlorinated solvents varied as follows: wind-blow sand > Davidson clay > farmland soil. Fluorobenzene was used as the internal standard in order to correct for the variation in the percentage recovery of these target analytes from the different soil matrices.

### Flow-injection extraction photometry using conductometric phase recognition

*Hanghuli Liu and Purnendu K. Dasgupta, Department of Chemistry and Biochemistry, Texas Tech University, Lubbock, TX 79409-1061, USA*

A flow-injection extraction detection system based on simultaneous determination of absorbance and conductance was described. The absorbance is read radially on a 300 μm bore PTFE tube with an optical aperture of ~0·5 mm, resulting in an effective detector volume of ~60 nl, small enough to resolve signals for each aqueous or organic segment. Consequently, the analytical signal can be obtained directly from the response of organic segments and the phase separation is not necessary.

The conductance measurement probes are located immediately after the optical aperture. Absorbance and conductance are respectively measured by light emitting diode (LED) based photometry and bi-polar pulse conductometry using a single personal computer for the operation of the dual detectors, data acquisition and processing. The phases are discerned based on the conductance signal and the analyte concentration is monitored by the absorbance signal. Accurate and reliable phase recognition is achievable with conventional T-segmentor and peristaltic pumping.

When the system is used to determine C-12 linear alkylbenzene suphonate (LAS), an anionic surfactant, by ion-pairing with methylene blue and extraction into chloroform, a linear response is observed in 0–2·5 ppm range. The limit of detection (LOD) for a 65 μl injected sample is 0·03 ppm; in comparison, the standard manual method that uses several hundred ml sample for extraction reports an LOD of 0·025 ppm.

### Flow injection immunoassay using a magnetic separator and immunomagnetic particles

*Ninglan Liao, Lachat Instruments, 6645 West Mill Road, Milwaukee, WI 53218-1239, USA*

Magnetic separation has been widely applied in enzyme linked immunosorbent assay (ELISAs). However, there has been little success with the method. Disadvantages include the time taken and poor reproducibility. Real time automated in-line instruments for immunoassay are anxiously awaited by analytical laboratories. Even though sequential injection immunoassay utilizing immunomagnetic beads has been proposed by the Ruzicka research group, the method introduced interference from the sample matrix into the results and limited this technique in testing real world samples.

In this novel work, a magnetic separator and immunomagnetic particles have been successfully used with flow injection immunoassay (FIIA) for enzyme linked immunosorbent assays (ELISAs). The optimized parameters of electronic magnetic separator (EMS) on FIIA were found by studying the relationship of flow rates and magnetic field strengths with the separation performance of magnetic particles coupled antibodies. The retention time for retained and unretained peaks can be simply controlled by the time of switching EMS on to off using software. This work created a very flexible real time automated FIIA analyser for the user. The analyser has the function to separate the enzyme-conjugate and antigen (analyte) combined with antibody coupled on magnetic particle from free enzyme and sample matrix in ELISA. It is able to measure a purified analyte without interruption of interference from the matrix. The applications of the technique were discussed.

### Identification of nicarbazin by near infra-red reflectance spectroscopy

*Keith B. Bradfield and Robert A. Forbes, Eli Lilly and Company, Clinton Laboratories, PO Box 99, Drop Code C22K, Clinton, IN 47842, USA*

Near infra-red reflectance spectroscopy (NIRS) was evaluated as a method for identification testing of nicarbazin granulated (N,N′-bis(4-nitrophenyl) urea, compound with 4,6-dimethyl-2(1H)-pyrimidinone (1:1) (CAS Number: 330-95-0); affixed to corn cob grits with polyethylene glycol) raw material in a pharmaceutical quality control laboratory. Replicate spectra of 15 lots were acquired over multiple days by analysts over a wavelength range of 1100 nm to 2500 nm using a NIRSystems (a Perstorp Analytical company) model 5000 instrument equipped with a two metre long fibre optic reflectance probe (the Smartprobe). Using statistical experimental design, the method parameters causing variation in the NIRS spectra of nicarbazin samples were determined. Once these critical parameters were discovered, the library was validated for ruggedness with respect to the critical parameters. The identification method was validated for selectivity by predicting the identity of 10 lots of nicarbazin not originally used in the validation set, and challenged for capability of discriminating other similar raw materials. The nicarbazin NIRS identification assay replaced a labour-intensive high performance liquid chromatography (HPLC) identification assay, helping to meet the British Medicines Control Agency requirement for testing every container (100% container testing) of a raw material lot for identity, without significantly increasing laboratory workload or prolonging raw material lot approval times.

### Chemical identification of incoming raw materials before it is a problem

*Paul J. Brimmer, Perstorp Analytical Inc., 28A Mimosa Street, Oatley, NSW 2223, Australia, and Denise E. Root, Perstorp Analytical, Inc., 12101 Tech Road, Silver Spring, MD 20904, USA*

One hundred per cent inspection of incoming raw materials is a requirement of ISO9000. This requirement places extreme burden on shipment receivers, as well as the quality control laboratory. Costly demurrage charges are accrued due to the extensive testing performed on every raw material.

Near-infra-red (NIR) spectroscopy offers a rapid, accurate pass/fail verification technique for the analysis of incoming raw materials. With results being obtained in under one minute, this nondestructive method requires no sample preparation or solvents.

This paper described the development and implementation of a qualitative NIR test method. Particular attention was focused toward the sampling requirements for the designed area (i.e. laboratory, receiving area), instrumentation employed and sampling constraints.

### Model system for noninvasive near-infra-red spectra of human tissue

*Jason Burmeister and Mark Arnold, Department of Chemistry, University of Iowa, Iowa City, IA 52242, USA*

The ability to measure blood glucose levels noninvasively is dictated by the signal-to-noise ratio (SNR) of near-IR spectra collected through the human body. The predominant features in noninvasive human spectra correspond to water and body fat which strongly absorb near-IR radiation. The authors have successfully developed a model system to simulate noninvasive human spectra. Layers of bovine fat and aqueous buffer solutions are combined within the sample compartment of a modified Nicolet 740 spectrometer. The corresponding near-IR spectra are similar to noninvasive human spectra in terms of absorbance features and spectral noise levels. This model system allows critical experimental parameters, such as optical throughput and fat layer thickness, to be investigated in a controlled and reproducible manner. Details of this model system, as well as results from an analysis of the effects of incident radiation intensity on the limit of detection for glucose, were detailed in this presentation.

### Extraction of fat and naturally occurring phospholipids for fatty acid profiling in food products

*Lori A. Dolata, Natalee Beitko and Athos C. Rosselli, Suprex Corporation, 125 William Pitt Way, Pittsburgh, PA 15238, USA*

This paper described routine extraction of fats and naturally occurring phospholipids from food products by SFE using both modified and unmodified carbon dioxide. The current AOAC fat extraction methodologies include both crude fat and total fat methods. Data were presented comparing SFE to these conventional techniques. For example, a statistical correlation between SFE and the conventional crude fat method using freon for the extraction of aromatic oils from freeze-dried and spray-dried coffee was presented. In addition, results obtained through SFE of fat from pet foods were compared to those from acid hydrolysis, a total fat method. The difference between crude fat and total fat is that total fat includes phospholipids which are tightly bound within the matrix and normally quite difficult to extract. The steps necessary in developing these SFE methods for the automatic extraction of both fats and phospholipids were described in detail.

### Rapid, automated sample preparation for pesticide residue analysis in high fat matrices

*Athos C. Rosselli and Lori A. Dolata, Suprex Corporation, 125 William Pitt Way, Pittsburgh, PA 15238, USA*

Large amounts of chlorinated solvents are used to clean up extracts from high-fat matrices prior to analysis by GC-ECD or GC-MS. These clean-up steps are necessitated because of the propensity of many extraction fluids, including supercritical carbon dioxide, to co-extract triglycerides, sterols and fatty acids from these matrices in addition to the pesticides of interest. Since these co-extractable materials are present in much greater amounts than the pesticides, time consuming and solvent-intensive clean-up steps are required prior to analysis, in order to achieve acceptable limits of detection.

The authors described the use of a novel supercritical fluid, fluoroform ($CHF_3$), as a means to selectively extract pesticides from rendered chicken fat, cocoa beans and other matrices. The use of fluoroform for this purpose is more effective than several other existing techniques, such as the use of activated alumina to retain fat in the extraction vessel. When this technique is employed for samples with pesticide concentrations at the ppb or ppt level, the small amount of fat which is not retained by the alumina is sufficient to interfere with quantitation, although the technique does work well at higher concentration levels. The use of fluoroform overcomes this problem, because the amount of fat (chicken fat, for example) extracted by fluoroform is about 100 times less than that extracted by carbon dioxide. The authors were thus able to directly inject fluoroform extracts into a GC-ECD or GC-MS without additional clean-up to obtain quantitative recoveries for the extraction of 21 organochlorine pesticides at trace levels. The authors also reported on the effect of methanol modified fluoroform, and on the comparative effectiveness of fluoroform versus other selective-extraction methodologies.

### Development of authomated SFE methods for dry, semi-wet and wet pet foods

*Natalee Beitko, Victor G. Danielson and Anita Cardamone, Suprex Corporation, 125 William Pitt Way, Pittsburgh, PA 15238, USA*

The authors described the steps used to develop a successful Supercritical Fluid Extraction (SFE) method for the quantification of fats and oils in a variety of pet food products. Current methodology commonly relies on exhaustive extraction with liquid solvents such as chloroform, hexane, petroleum ether, etc., in a process which can take up to 12 hours. SFE not only greatly reduces this time, but it also eliminates most of the liquid solvents by using supercritical carbon dioxide. This paper addressed the selection of the appropriate extraction parameters which include pressure, temperature, and the addition of modifier, and data were presented to illustrate the effect of each of the parameters. The optimization of extraction time was also discussed with the use of extraction rate curves to indicate the optimum balance between recovery and length of extraction.

Using SFE, the percentage fat in pet food products is determined gravimetrically by either calculating the amount of fat lost from the sample or the amount of fat gained in a collection vial. In either case, the amount of water in the pet food samples must also be taken into consideration when making these calculations. The method details associated with both pre-extraction and post-extraction strategies designed to handle the effect of water in pet food were discussed, especially with respect to operation in a routine QC or process control laboratory.

### Automated hydrazine free nitrogen measurement in water and wastewaters

*Bhupendra R. Patel and Camille Murray, Bran+Luebbe, Inc., 1025 Busch Park Way, Buffalo Grove, IL 60089-4516, USA*

Nitrogen measurements are very important in water and wastewater treatment industries. The forms of nitrogen are critical to monitor the denitrification process. Excessive amounts of oxidized nitrogen in drinking-water can cause illness such as methemoglobinemia in infants. A limit of 10 mg/l nitrate as nitrogen has been imposed on drinking water to prevent this disorder.

Nitrogen from $NO_2$, and $NO_3$ oxyanions is a regulated parameter in NPDES (National Pollution Discharge Elimination System) and drinking-water under SDWA (Safe Drinking-Water Act). Under the approved test procedures, nitrate nitrogen can be analysed by the hydrazine reduction, using either manual or automated methods. However, rising concern about the carcinogenicity of hydrazine has forced a change for an alternative method for nitrate reduction. It is reported that high doses of hydrazine induce pulmonary tumours in mice. In 1991, USEPA approved the cadmium reduction method for nitrate nitrogen analysis. The same reduction technique is presented in EPA/600/R-93/100 of August 1993 under Method 353.2.

The Bran + Luebbe multitest manifold for environmental testing is used for different analyses by changing reagents. The cadmium reduction coil must be physically disconnected while running other methods. To eliminate the disconnection procedure, a directional loop valve is incorporated on the multitest manifold. This paper discussed the detailed information and data on nitrogen analysis from nitrate and nitrite. The information presented in this paper can be useful to industrial and municipal water and wastewater analysts, plant operators, and regulatory agencies who are interested in alternative test procedures for nitrogen measurement.

### A new method for on-line determination of trihalomethane and cyanogen chloride content in treated drinking water using membrane introduction mass spectrometry (MIMS)

*Scott Bauer, Timothy Griffin and Jan Bauer, MIMS Technology Inc., P.O. Box 60129, Palm Bay, FL 32906, USA*

Trihalomethanes (THM) are formed to varying degrees in the chlorination treatment of drinking-water. These compounds are hazardous to human health and their levels in drinking-water are strictly controlled by environmental regulation. Cyanogen chloride (CNCl) has recently been identified as another hazardous by-product of certain chlorination treatment processes and it will probably be regulated in the near future.

Traditional analysis methods approved by the Environmental Protection Agency (EPA) for the determination of THMs in drinking-water rely on periodic spot checks. Samples are removed from the treated water stream and shipped to an external water quality laboratory for analysis. These spot checks may be performed as infrequently as twice per year.

The periodic spot checks on water quality rely on the assumption that the condition of the water treatment process is the same at all times. The current testing method also assumes that the results obtained on water samples shipped to an external laboratory accurately reflect the condition of the bulk water from which the sample was drawn. This assumption is being challenged with increasing experimental evidence demonstrating that volatile organic samples degrade during shipment and storage and rarely reflect the true nature of the reservoir from which they were drawn. This knowledge has spawned a rapidly growing industry dedicated to field screening and on-site analysis instrumentation and techniques.

The experiments described in this paper demonstrated the utility of membrane introduction mass spectrometry (MIMS) as applied to the real-time monitoring of water treatment process streams for the determination of THM and CNCl levels on-line. Use of a MIMS monitoring system provides a much more accurate picture of the water treatment process over time by providing a continuous record of temporal variations in THM and CNCl levels in the process stream. The ability to assess water quality in real time allows the water treatment facility the ability to take corrective action on out-of-control processes before tainted water can reach storage reservoirs where remediation is difficult or impossible.

Data was presented to demonstrate the utility of MIMS in the determination of trihalomethane and cyanogen chloride levels in water in real time with a high degree of accuracy. Repetitive analyses demonstrate average %RSD values of 2.5% and recoveries near 100%. Detection limits established for THMs in water using this MIMS system are 200 parts-per-trillion. The detection limit for CNCl is 1 part-per-billion. These results are obtained through direct injection of the water sample into the MIMS system with no sample preparation or pre-concentration required. Matrix interferences are non-existent.

The utility of the on-line MIMS system is further enhanced by the ability to screen for the presence of 72 volatile organic compounds in parallel with the THM and CNCl analysis. Data were presented that demonstrate the ability to detect the presence of the listed volatile organic compounds including EDB and DBCP below the maximum contamination level (MCL). Detection limits for most of the 72 listed volatile organic compounds are in the mid parts-per-trillion range. Validation data obtained from two independent laboratories were also presented.

This method has been submitted to the EPA for approval in on-line monitoring and off-site laboratory analysis. Use of this method in the laboratory can increase volatile organic analysis productivity by a factor of 10 at a fraction of the cost of traditional purge and trap GC techniques.

### A hand portable gas chromatography/ion mobility spectrometry instrument for Chemical Weapons Convention (CWC) verification

*N. S. Arnold, D. L. Hall, FemtoScan Corporation, 747 E. South Temple, Suite 101, Salt Lake City, Utah 84102, USA; R. Wilson, Graseby Ionics Ltd, Odhams Trading Estate, St. Albans Road, Watford, Herts WD2 5JX, UK; W. H. McClennen, H. L. C. Meuzelaar, Center for Micro Analysis and Reaction Chemistry, 214 EMRL, University of Utah, Salt Lake City, Utah 84112, USA; and A. P. Snyder, US Army ERDEC, Attn: SMCCR-RSL, Aberdeen Proving Ground, MD 21010-5423, USA*

The development of handheld chemical weapons (CW) detection technology for point use by US Military and NATO military forces over the last 10 to 12 years has centred around development of direct sampling Ion Mobility Spectrometry (IMS) instruments including the Chemical Agent Monitor (CAM) developed by Graseby Ionics. Although IMS is a valuable technology in its own right, recent developments have illustrated that high speed Transfer Line Gas Chromatography (TLGC) may greatly enhance the interferant rejection of IMS systems. It has been further shown that this technology may be readily coupled to a high speed micro volume trap and desorb unit to enhance detection limits into the mid ppt range. A new hand portable TLGC/IMS system with an add-on trap and desorb preconcentrator has been built. It is based upon Graseby Ionics CAM and FemtoScan Enviroprobe Automated Vapour Sampling (AVS) TLGC technologies and is called the EVM II (Environmental Vapour Monitor).

The integration of these three components into a hand portable device capable of operating for several hours without any external support (such as power supplies, etc.) is a necessary package for on-site inspection under the CWC. The EVM II has been shown to detect CW agents, precursors and other organic compounds at low ppb levels using direct air sampling and a short ($<20s$) GC preseparation. Low ppt levels using a trap and desorb preconcentration may be obtained in analytical cycles of less than two minutes. In both modes the instrument offers a level of analytic specificity surpassed only by hyphenated laboratory techniques (for example GC/MS).

### Environmental monitoring using laser ablation ICP-MS

*A. Raith, P. T. Sigsworth and R. Henry, Fisons Instruments Elemental Analysis, Ion Path, Road three, Winsford, Cheshire CW7 3BX, UK*

Environmental contamination requires close monitoring, particularly for toxic and heavy metal pollution. Marine life and ferromanganese crusts, for example, can be analysed as a record of long-term pollution in sea water, and life science studies can help as an indicator of general contamination.

Solution techniques require dissolution of the samples, during this sample preparation method all information about zonation, element distribution between growth-bands etc., are lost. This information can only be obtained by direct analysis of the solid sample. In this study, LA-ICP-MS measurements were performed with a new UV laser ablation system, the UV MicroProbe, which offers spatial resolution of craters of less than 10 m diameter, leading to detailed elemental mapping capabilities for trace levels.

### Simultaneous monitoring of pH, $CO_2$ and $O_2$ using an optical imaging fibre

*Brian G. Healey, Jane A. Ferguson and David R. Walt, The Max Tishler Laboratory for Organic Chemistry, Department of Chemistry, Tufts University, Medford, MA 02155, USA*

Clinical, biological and environmental systems often require simultaneous monitoring of multiple analytes due to their interrelated dynamics. Monitoring can be achieved by sensors fabricated using optical imaging bundles. The authors presented the fabrication and performance of a multi-analyte sensor that allows pH, $CO_2$ and $O_2$ to be monitored simultaneously with rapid response to all three analytes. Sensing elements are fabricated by covalently immobilizing fluorescent indicators within polymer matrices. Photopolymerization is achieved by discrete illumination at the proximal end of the fibre and results in the formation of distinct regions of analyte-sensitive polymer at the distal end.

### Automated capillary electrochromatography

*Maria T. Dulay, Chao Yan, Richard N. Zare, Department of Chemistry, Stanford University, Stanford, CA 94305-5080, USA and David J. Rakestraw, Sandia National Laboratories, Livermore, CA 94551, USA*

Since its first introduction as a separation technique, capillary electrochromatography (CEC), which is based on the advantage of combining the high efficiency of capillary zone electrophoresis (CZE) with the high selectivity and universality of liquid chromatography, has been utilized to analyse neutral compounds that are not separable by CZE. The use of CEC as an analytic technique has not become routine, however, because of problems associated with reliability of separations, reporducibility of separation runs, and the need to automate its use.

In this study the routine application of CEC was demonstrated by carrying out the separation of a mixture of neutral compounds with packed capillary columns incorporated into a commercially available capillary zone electrophoresis instrument. Fused-silica capillaries were packed with micron-sized particles (for example 3-mm octadecylsilica). A mixture of several neutral compounds was separated into its components with an average efficiency up to 181 000 plates/m in less than 10 minutes. Hundreds of consecutive runs were performed over a period of weeks from which it is concluded that the reproducibility of the capacity factor is better than 2% and that CEC separations can be achieved in a reliable manner.

### A completely automated system for the analysis of polymeric materials by pyrolysis GC

*Thomas P. Wampler and Woodford A. Bowe, Jr., CDS Analytical, Inc., P.O. Box 277, 7000 Limestone Road, Oxford, PA 19363-0277, USA*

Pyrolysis-gas chromatography has been demonstrated to be a simple and inexpensive means for the analysis of polymetric systems including textile fibres, paints, papers and coatings, biopolymers, plastics any many similar materials. Filament pyrolysis, using a strip or coil of resistive wire to heat the sample, offers the widest range of temperature and heating rates, but has been difficult to automate, due to the complexity of sample introduction. This paper describes a completely automated means of delivering up to 40 samples in quartz sample tubes into a coil filament pyrolyzer for unattended analysis of multiple runs.

The automated system includes a sample feed mechanism, pneumatics to place the pyrolysis chamber on-line with the GC injection port, automatic GC start, sample removal and pyrolysis chamber clean runs between analyses.

Examples of a variety of polymeric materials, from pure synthetic polymers to complex systems such as automotive paints and clothing fibres, were given to demonstrate the range of applications for automated PY-GC in analytical and QC laboratories.

### Organizing AA and ICP data for regulatory compliance, reporting, and archiving

*William B. Barnett and Peter Seferovic, Perkin-Elmer Corp., 761 Main Avenue, Norwalk, CT 06859, USA*

Modern, automated atomic absorption and ICP instruments use methods to control instrument settings, define the analytical sequence, and control the calculation of concentrations. Information must be supplied describing each batch of samples being analysed including sample IDs, weights, and dilutions. During analyses raw signals are generated and a variety of values are calculated including sample concentrations, calibration curves, and statistics. In many cases knowing the sequence of measurements is important to verify data quality based on the periodic analysis of QC samples. Many laboratories require that all of this information be saved to document the analyses and allow for post-analysis processing, reporting, and auditing.

Organizing the large amount of information associated with a series of AA or ICP analyses requires careful thought. Database tools provide a mechanism for saving the information in a series of linked tables. The problem for the system designer is deciding which data to save, how the different data types should be linked to each other, and how the information should be organized so that it can be accessed in an optimum way.

For the authors' WinLab series of AA and ICP instrument control software products, a number of objectives that the results library must meet including the following were defined:

(1) It must be compatible with a large number of commercial, off-the-shelf database and reporting tools. This allows a laboratory to customize queries and reports to meet its individual needs if those produced by the reporting tools included with the software are inadequate.

(2) It must accommodate single element AA, multielement AA, and multielement ICP data within the same structure. Accommodating data from these different techniques recognizes that much of the data are common between the different instruments and allows the laboratory to develop one set of data handling procedures and apply them to multiple products.

(3) It must include audit trail information such as details of the method used, calibrations performed, raw data collected, calculated concentrations, and error messages. Information of this type is important since many laboratories must comply with the requirements of quality systems such as GLP, GALP, and ISO 9000.

(4) It must store the raw data required for post analysis recalculations and data review.

To meet these objectives the Borland IDAPI database engine was used by the WinLab software products to write the data in a Paradox for Windows relational database format. The tables are organized to accommodate the manner in which the instruments use input information (from methods and sample information files) and generate data. Data for batches of samples are stored as named data sets. Tables are included to save sample description information, information from each replicate measurement, mean data for each determination, calibration curves, an error log, and a set of tables containing the complete method used to perform the analyses. A series of indices link the data for each determination.

### Automatic long term quality control and diagnosis of ICP-OES with dedicated software

*O. Samuel, Instruments S.A., Division Jobin-Yvon, 16/18 Rue du Canal, 91165 Longjumeau, France*

In the whole analytical procedure, from the sample to the results, the user of high frequency inductively coupled plasma (ICP) has to be sure that the instrument provides good analytical performance at least equal to previous techniques.

It is therefore necessary to provide simple experiments that can be applied on commercially available ICP systems. This motivated the development of a set of tests and a software based on previous work on drift, on the diagnosis using the ratio of the intensity of an ionic line to that of an atomic line and to a procedure of diagnosis of the ICP. This procedure is fast and easy to perform so that there is no time penalty in conducting a periodic check.

In addition to argon, a limited number of elements are used: Ba, Mg and Zn. Simple experiments allow the ICP user to verify practical resolution, efficiency of energy transfer between the plasma and the aerosol, degradation of collimating optics, nebulizer efficiency, repeatibility, long term reproducibility and limits of detection.

This procedure has been tested by several Jobin-Yvon customers. Results were presented to describe the main causes of malfunctions and to assess the analytical performance over a long period of time.

### Direct and near real-time determination of metals in air by impaction-graphite furnace atomic absorption spectrometry

*Suneetha Indurthy, Joseph Sneddon, Mark Smith, Yong Ill Lee and Anupama Deval, Department of Chemistry, NcNeese State University, Lake Charles, LA 70609, USA*

Atmospheric and industrial pollution caused by metals or metallic compounds in air is a growing environmental concern. Therefore a system that can determine metals directly in air or on a real or near real-time basis is of great need and interest.

This work investigated a single stage impactor used for sample introduction and collection in combination with a graphite furnace for quantification by atomic absorption.

The principle of this single stage impactor, the design, operating characteristics and a preliminary evaluation of this aerosol collection system were presented. The theoretical and experimental factors that effect collection efficiency such as nozzle width, particle size, and flow rate are under further investigation.

### Facilitating chromatographic control on centralized computer systems

*Brett M. Jackson, Beckman Instruments, Laboratory Automation Operations, 90 Boroline Rd., Allendale, NJ, 07401, USA*

In laboratories with centralized chromatography data systems, laboratory managers need to do more than just acquire raw data from their chromatographs. In R & D labs, automatic documentation of instrument settings is desired. In QC labs, centralized control is coveted. The exchange of information between instruments and computers allows laboratories to better address regulatory issues, be more efficient, and experience fewer transcription errors.

Microprocessor based instruments have the flexibility to address some of the above needs. Software standards have been established which facilitate the exchange of information between various vendor chromatography data systems. The problem is that most laboratories have instruments from diverse manufacturers. Each instrument may provide equivalent functionality, but the means for electronic exchange of information is peculiar to each system. If one desires to control or simply record settings of an instrument for an analysis, a custom solution is required.

What is needed is a means to facilitate control of or communications with various instruments. The method should not require major software overhauls in order to support different instrument configurations. This feature would speed up the instrument control development process. Additionally, the approach needs to provide flexibility in updating code embedded in instrument interface or computer as bugs are discovered, instrument configurations change or instrument firmware is enhanced.

Requirements for a chromatograph interface to address these goals were discussed in this paper.

### A remote, *in-vivo*, fibre-optic spectrofluorometer for human skin and photodynamic therapy measurements

*Raymond Kaminski, Karen J. Rubelowsky and Kathleen M. O'Brien, Instruments S.A. Inc., 3880 Park Ave., Edison, NJ 08820, USA*

There are many absorbing chromophores in normal human skin that exhibit intrinsic fluorescence. The fluorescence characteristics of these individual chromophores such as tryptophan, elastin, collagen and NADH can be monitored to determine the condition and structure of skin. The spectral profile of the fluorescence and skin remittance can evaluate the degree of dryness and wrinkles, damage due to UVA and UVB, pigmentation, skin turnover rate, and elasticity properties.

Another technique that would benefit from a remote fibre-optic spectrofluorometer involves Photodynamic Therapy (PDT). In this technique, the fluorescence probe is excited with light from the fibre-optic bundle. The excitation wavelength is optimized for the specific probes. Singlet oxygen is produced which subsequently decreases the tumour size. This is an alternate method for management of the tumour when surgery is not possible. Photodynamic Therapy is a viable method for managing tumours, preventing growth, and ultimately destroying the cancerous cells.

These applications were discussed to demonstrate how a remote, *in vivo*, fibre-optic spectrofluorometer can be a valuable tool for the research scientist involved in characterizing skin and cancerous tumours.

### Flow injection analysis with near infra-red detection

*Mauricio S. Baptista and Chieu D. Tran, Department of Chemistry, Marquette University, P.O. Box 1881, Milwaukee, WZ 53201-1881, USA*

A near infra-red (NIR) spectrophotometer based on acoustic optic tunable filter (AOTF) has been developed as a new and universal detector for flow injection analysis (FIA). In addition to being compact and all solid state, this AOTF based instrument is very sensitive, has high resolution and can be rapidly scanned. The latter advantage means that it is uniquely suited as a detector for FIA: it can rapidly record the whole NIR absorption spectrum of a mixture passing through the FIA flow cell. This detector was tested in both static determinations (no flow) and FIA conditions. Subsequent treatment of the recorded spectra with partial least squares makes it possible to use the FIA to determine, a variety of systems including, the determination of trace amounts of water in chloroform (LOD = 13 ppm and RMSD = 0·002%) and the determination of trace amounts of water (LOD = 80 ppm and RMSD = 0·006%) and benzene (LOD = 124 ppm and RMSD = 0·018%) in ethanol (the simultaneous determination of both components in ethanol provided RMSD = 0·015 and 0·033% for water and benzene, respectively). Because all organic compounds absorb light in the near infra-red region, this AOTF based near infra-red detector can serve as a universal detector for FIA, and consequently, helps expand the applications of the FIA techniques to other areas which are not possible otherwise. Specifically, there is not a method currently available that can be easily coupled with FIA instrument for the sensitive and simultaneous determination of the concentrations of two or more components without any sample pretreatment.

### The analysis of PPB metal hydride impurities in specialty gases by GC/RGD

*William M. Geiger and Carol J. Meyer Merlin MicroScience, Inc., P.O. Box 19528, Houston, TX 77224, USA*

The analysis of metal hydride impurities in speciality gases is of concern to semiconductor manufacturers. These impurities can cause deleterious effects as impurities in many deposition processes when present even at low ppb concentrations. The Reduction Gas Detector has been found to have ppb sensitivity to these hydride impurities and has been used in conjunction with a gas chromatographic system to separate and quantitate silane, phosphine, and germane impurities in bulk arsine.

The mechanism of detection of the RGD is the selective reaction of reducing gases with a mercuric oxide bed followed by the detection of the freed mercury by means UV absorption. Because of the strong absorption of mercury, this detector is extremely sensitive. However, due to the reaction chemistry of the detection scheme, complex interactions can occur between components which are not separated prior to the reaction in the mercuric oxide bed.

### Automated gas sampling system for the analysis of multiple cylinders

*John S. McWilliams and Jeremiah D. Hogan, Texas Instruments, Inc., P.O. Box 650311 M/S 301, Dallas, TX 75265, USA*

Many types of laboratory samples lend themselves well to the automation of the steps necessary for their preparation or analysis. Cylinder gas samples are a different matter. The physical size of the sample container, and the necessity to ensure a closed sampling system, require a considerably different approach than those used in the analysis or more traditional samples.

In addition to the difficulties in obtaining a representative sample of a cylinder gas, another challenge faces the manager of any laboratory today: the need to quickly and accurately integrate laboratory data with other business systems.

This work demonstrated the development of a system to allow the unattended sampling and analysis of up to eight cylinder gas samples. A customized sampling system allows the connection of multiple instruments to a single sampling point, allowing the simultaneous determination of multiple components of interest. Customized software written using industry-standard tools allows the rapid acquisition, processing, and further integration of laboratory data with other information systems.

### A quality control crisis: the method detection limit versus the practical quantitation limit

*Michael J. Herdlick, Aqua Tech Environmental Laboratories, Inc., 6878 South State Route 100, Melmore, OH 44845, USA*

One of the great mysteries in the world of environmental chemistry is the concept and use of the Method Detection Limit (MDL) and Practical Quantitation Limit (PQL). These two mathematical calculations are interpreted in various ways by different individuals in the laboratory community. Nearly all of the US EPA methodologies discuss the purpose and the practicality of the MDL and the PQL, but what do these two respective values actually mean? Are these numbers simply statistical calculations independent of any analytical chemistry concerns or do these limits actually support the generation of legally defensible data in the typical environmental laboratory.

The debate over the issue of the Method Detection Limit and the Practical Quantitation Limit has led to a quality control crisis with little or no hope of being resolved anytime in the near future. This paper focused on:

(1) The equation used to calculate the Method Detection Limit and the Practical Quantitation Limit.
(2) The mathematical theory behind the MDL and PQL.
(3) The raw data from a number of different analytical methods used to calculate these values.
(4) A summary of quality control pitfalls that occur based on numbers generated as MDL or PQL.
(5) An alternative approach for calculating numerical limits based on respective analytical methods.

The environmental community continues to ponder the use and the goals behind the Method Detection Limit and the Practical Quantitation Limit. With the many existing regulatory programmes, one can be in a terrible quandary concerning these theoretical concepts, especially when a client merely wishes to know if their respective analytical result is high, low, safe, or hazardous. This paper was of particular interest to those dealing with regulatory issues such as detection limits and quality control conerns.

### LIMS software validation and document management

*Robin J. McDaniel, RJM Laboratory Services, R&D Div., 607 Douglas Ave., Elgin, IL 60120, USA*

The modern laboratory is a complex multi-faceted organization where critical software and systems must be carefully protected to assure functionality while maintaining the quality of the end result: accurate experimental data and high confidence information.

To achieve this, the existing LIMS vendors have taken good measures to provide software which should include functions to keep track of the samples and results in the laboratory. However, this is no longer good enough since the complexities of laboratory data management in a regulatory environment (as well as others), demand that higher quality control and systems standards be applied by the users to all laboratory software used in the preparation of analytical results.

A rigorous validation methodology that addresses functional requirements, design specifications, test plans, and acceptance documents, will by its nature, create hundreds of documents. Each one needs to be reviewed, approved, and distributed to all members of the validation community of the laboratory. Only by incorporating the ideas proven in the document management arena, and applying them creatively to the laboratory can this large task be accomplished.

The current concepts of: Validation Planning, Procedures Development, and Version Control, can be incorporated with Document Control techniques to assist in the validation requirements of the laboratory.

By utilizing LIMS validation/document management software, the success of the project, and the time required to validated the system, can be optimized and many benefits of good management can be achieved.

### Air monitoring by membrane introduction mass spectrometry: going beyond volatile organic compounds

*P. H. Hemberger, M. E. Cisper and A. W. Garrett, Chemical Sciences and Technology Division, Los Alamos National Laboratory, Mail Stop J565, Los Alamos, NM 87545, USA*

Membrane introduction mass spectrometry (MIMS) is well-established for the analysis of volatile organic compounds (VOCs) in water and air. Semi-volatile organic compounds (SVOCs), because of their lower vapour pressures and lower diffusivity through the commonly used semi-permeable membranes, are not as amenable as the VOCs for analysis by MIMS. However, SVOCs have significant environmental importance and on-line or field analytical methods for these compounds would be a valuable tool The simplicity of MIMS would make it ideal for such applications if adequate detection limits are achieved.

The authors described the application of semi-permeable membranes for the analysis of SVOCs by MIMS. In these experiments the membrane probe is placed directly inside the ion source of a Finnigan GCQ Ion Trap Mass Spectrometer. The high boiling SVOCs are volatilized by heating the membrane in the ion source in a fashion similar to that described by Lauritsen; the SVOCs are subsequently ionized by either electron ionization (EI) or chemical ionization (CI) and injected into the ion trap for mass analysis. The external ionization source provided by the Finnigan GCQ provides several important features that contribute to the success of this method. The first is the ease with which it is interfaced to the membrane probe; the second is versatility in ionization methods, including the ability to use negative chemical ionization, which has been historically difficult in the ion trap; the third is the ability to reject unwanted species from the ion trap. Results for a variety of semi-volatile compounds with boiling points to over 300 °C were given. The use and selection of EI versus CI was discussed, and the utility of MS/MS for mixture analysis of SVOCs was demonstrated. Preliminary experiments indicate that detection limits will be in the low to sub parts-per-billion by volume for air samples.

### On-line monitoring of chemical and biochemical reactors by membrane introduction mass spectrometry

*R. G. Cooks, N. Srinivasan, P. S. H. Wong, N. Kasthurikrishnan and R. C. Johnson, Department of Chemistry, Purdue University, West Lafayette, IN 47907-1393, USA*

Membrane introduction mass spectrometry (MIMS) is a method for on-line, continuous and often very sensitive detection of organic compounds in water, air and less frequently, organic matrices. The method has the rapid response, high compound specificity and high sensitivity characteristic of mass spectrometry and the convenience of implementation of flow injection analysis methods of sampling and quantitation. While MIMS has been practised for environmental and process analysis for a number of years, increased recent interest in the technique has led to commercial interfaces.

This presentation covered several recent developments in membrane materials and interfaces. Comparisons were made between the performance of various interfaces and membrane types, with some emphasis going to the use of liquid as well as conventional polymer membrane materials. The exploration of membrane materials which are selective for particular classes of compounds was especially emphasized.

Photolytic reactions were used to illustrate the capabilities of MIMS in monitoring the course of chemical reactions and in deriving kinetic data on reacting systems. In similar vein, it was shown that biological fermentations can be monitored continuously over a long period and the information used to optimize the fermentation process, through feedback control.

Ultra-low level analysis was shown to be possible for volatile organic compounds using ion trap mass spectrometers and data on cyanogen chloride, peroxyacetylnitrate (PAN), and other compounds of environmental interest were presented to show the continuing relevance of this rapid method of chemical screening.

### Monitoring an oxidative stress mechanism at a single human fibroblast

*Stephane Arbault,[1] Paul Pantano,[1,2] Jeffrey A. Jankowski,[1,3] Monique Vuillaume,[4] and Christian Amatore[1], [1]Ecole Normale Supérieure, Département de Chimie, URA CNRS 1679, 24 rue Lhomond, 75231 Paris Cedex 05, France; [2]Department of Chemistry, Tufts University, Medford, MA 02155, USA; [3]Nalco Chemical Company, 1 Nalco Center, Naperville, IL 60563, USA; [4]Institut de Recherche sur le Cancer, UPR CNRS 42, 94801 Villejuif Cedex, France; and Ecole Normale Supérieure, Départment de Biologie, 46 rue d'Ulm, 75230 Paris Cedex 05, France*

Easily oxidizable substances inside human diploid fibroblast cell strains were monitored amperometrically with a platinized carbon-fibre microelectrode. The experiment involved positioning a microelectrode over a single biological cell, forcing the electrode tip into the cell via micromanipulator control, and measuring the transient current corresponding to the complete electrolysis of electroactive species released by the cell. A second series of experiments involved puncturing a hole into the cell with a micropipette and measuring the transient current corresponding to the complete electrolysis of electroactive species emitted by the cell with an electrode positioned above the cell. The selectivity of both amperometric measurements was demonstrated through the use of known hydrogen peroxide scavengers (added catalase, or intracellular peroxidase + added o-dianisidine) to the media bathing the cells. The abolition of the amperometric signal under these conditions suggested that hydrogen peroxide was the primary substance detected. The magnitude and the time course of the transient current measured implied that the hydrogen peroxide detected was not only that initially present in the cell before its membrane was pierced but represented mostly that from an oxidative stress response of the cell to its injury.

### *In vivo* imaging fluorescence spectroscopy for detection of damage to leaves by fungal phytotoxins

*Walter J. Bowyer,[1] Li Ning,[2] Larry S. Daley,[2] Gary A. Strobel,[3] Gerry E. Edwares,[4] James B. Callis,[5] [1] Dept. of Chemistry, Hobart and William Smith Colleges, Geneva, NY 14456, USA; [2] Dept. of Horticulture, ALS 4017; Oregon State Univ., Corvallis, OR 97331, USA; [3] Plant Pathology Dept., Montana State Univ., Bozeman, MT 59717, USA; [4] Botany Dept., Washington State Univ., Pullman, WA 99164, USA; [5] Dept., Chemistry, Univ. of Washington, Seattle, WA 98195, USA*

Imaging spectroscopy promises to be an important tool for computer diagnosis of diseases, insect damage, and nutritional requirements of plants. The authors are developing techniques for *in vivo* fluorescent and visible imaging spectroscopy. The *in vivo* fluorescence of chlorophyll is particularly promising for diagnosis of fungal infection. Evidence was presented that certain phytotoxins (especially triticone and pestalopyrone) interrupt the electron transport chain from the excited chlorophyll-protein complexes. This results in an increase in fluorescence relative to a healthy leaf.

To maximize the imaging of the damage and minimize variability in the chlorophyll concentration over the surface of the leaf, fluorescent images are recorded as a function of time. The images are then manipulated with a simple algorithm which compares steady state fluorescence to the maximum fluorescence for each pixel of the image. The resulting parameter is closely related to photosynthetic efficiency.

Four toxins isolated from fungal pathogens (pestalopyrone, hydroxypestalopyrone, triticone, and pestaloside) were injected into leaves of *Hibiscus*. Images were recorded as a function of time and dosage. While the *Hibiscus* were not sensitive to two of the toxins, the damage caused by triticone and pestalopyrone was obvious by fluorescent imaging 30 minutes after injection. After several hours, the leaves recovered from pestalopyrone damage. Triticone damage continued to increase for six hours and became visually obvious as a brown ring around the injection site.

Damage induced by the phytotoxins was shown to be detectable by fluorescent imaging much sooner than it is apparent by simple visual inspection. The technique not only has potential applications for computer diagnosis of fungal infection, it can also be applied as a rapid bioassay in the isolation of toxins from the fungi.

### Evaluation of different calibration strategies for ETV-ICP-MS

*Nancy J. Miller-Ihli and Rodney W. Fonseca, US Department of Agriculture, Beltsville Human Nutrition Research Center, Bldg 161, BARC-East, Beltsville, MD 20705, USA*

Electrothermal vaporization (ETV) is one of the sample introduction techniques that is currently used in inductively coupled plasma mass spectrometry (ICP-MS). This alternative technique to solution nebulization presents several advantages including: improved sensitivity, small sample requirements and the capability for direct solids analysis. Direct solid sampling greatly reduces sample preparation time, minimizes the risk of sample contamination, and reduces acid waste. Ultrasonic slurry sampling is a useful technique for direct solids analysis, allowing solid sample introduction using liquid sampling handling apparatus.

The purpose of this study was to evaluate different strategies and assess their usefulness for both slurries and sample digests. Three different calibration strategies were evaluated: (1) external calibration using aqueous standards; (2) method of additions; (3) the use of In as an internal standard. Slurry samples were introduced into the ETV cell using the ultrasonic slurry sampling (USS) technique. Oxygen ashing was employed in order to remove the organic matrix present in slurry samples. Oxygen ashing improves signal intensities as a result of increasing analyte transport. Matrix suppression effects due to high concentrations of matrix constituents were observed in some cases. When matrix suppression effects were present the method of external calibration provided low recoveries (average accuracy: $73 \pm 12\%$), therefore it was necessary to use the method of additions to compensate for these problems, providing an average accuracy of $108 \pm 13\%$. When matrix effects were absent, the external calibration method resulted in an average accuracy of $101 \pm 16\%$. The elements studied included: Mn, Ni, and Cu and the materials analyzed included: NIST SRM 1548 Total Diet, and SRM 1549 Milk Powder. Pd was used as a physical carrier. Benefits and limitations of this techique were discussed.

### Automatic polarity switching DC-Arc: a tool to augment multi-element analysis of solids

*Charles Hodges, A Kamenschikov and Gerhard Meyer, Thermo Jarrell Ash, 27 Forge Parkway, Franklin, MA 02038, USA*

DC-Arc continues to be an important spectroscopic technique for solid samples. For samples where acid dissolution is impractical or detrimental to achieving low detection limits, the DC-Arc can sample powders, briquettes, wires, chips, and other conductive solids in their original form without further handling. Several improvements have been made to the DC-Arc to further enhance its already widely accepted utility. The most notable change has been the way the light from the arc is measured. The photomultiplier had been the detector of choice ever since it replaced photographic plate technology over 30 years ago. However, the photomultiplier instruments lacked the complete spectral coverage that photographs provided. The marriage by TJA of the CID (Charge Injection Device) detector with the DC-Arc several years ago has created an exiting new instrument the provides both complete wavelength coverage and fast, precise determinations.

Another innovation by TJA in the use of DC-Arc has been the ability to switch the polarity between the sample and the counter electrode. For many kinds of geological and oxide powders, better sensitivity for elements such as selenium, arsenic and bismuth can be achieved by making the sample the anode during a portion of the analysis. Other desired elements can still be determined at published detection limits by switching the sample from being the anode to the normal configuration where the sample is the cathode, all during the same analysis. This presentation described the polarity switching in greater detail, and highlighted its use for the determination of elements in a variety of matrices.

### Analysis of multispectral image data utilizing a neural network based on adaptive response theory

*Ying X. Noyes and Scott E. Carpenter, Analytical Chemistry Laboratory, Chemical Technology Division, Argonne National Laboratory, 9700 South Case Avenue, Argonne, IL 60439-4837, USA*

The technique for automatic target recognition using airborne imagery is a key component in both military defence and arms control. This work presented a novel technique that provides automated, accurate and fast target recognition, regardless of target size (altitude) and orientation relative to the flight line. Data used in this study were collected by the Daedalus airborne multispectral scanner (AMS) system (Daedalus Enterprises, Inc., Ann Arbor, MI). This system can record images for up to six spectral channels simultaneously. A total of 10 bands covering the spectral range from 420 nm to 14 000 nm are available. Data were collected from the Norfolk Naval Shipyards (Virginia), Chesapeake Bay (Virginia), Duck Creek (North Carolina) and Camp Lejeune (North Carolina), thus naval ships were the targets to be identified.

A heuristic algorithm was developed to separate dock areas from land areas, then images from multiple spectral bands were combined and used simultaneously to isolate ships from docks. After individual ships were separated from land and docks, the region labeling technique was employed for segmentation. Features were then extracted utilizing invariant moments feature vectors that provide the size and orientation invariant description. These feature vectors are the input to the neural network. The ART2A neural network is a self-organizing system that has the ability to recognize familiar patterns, and more importantly, it also is capable of identifying and learning unfamiliar patterns *dynamically*. This means that if a new, unknown target is found, it will be recognized as such, and the system will dynamically create a new class for later recognition use. Due to the simplicity of this algorithm, it is computationally rapid, which makes this technique possible to be incorporated into an on-line system.

A total of 94 ships were included in the analysis. Ships were well identified and in particular, moving ships were distinguishable from the docked ships. The cross validation results showed the overall pattern recognition accuracy is 96·8%. The ability of identifying and creating new class(es) was also proven. By combining innovative image preprocessing procedures with a state-of-the-art pattern recognition model, this work represents a highly effective technique for on-line, automated and fast identification of ships in multispectral images.

### Calibration with controlled uncertainty

*Werner Haesselbarth, Bundesanstalt fuer Materialforschung und-pruefung (BAM), Referat I.01, D-12200 Berlin, Germany*

Calibration forms an essential component of most instrumental analysis procedures. Further, it is calibration that constitutes the link between reference materials and analysed samples that is necessary for traceability of analytical results. The uncertainty of calibration is therefore a key element in the overall uncertainty of an analytical procedure. At present, though much labour has been invested in establishing the best fit regression curve for various statistical models, little work has been done on the evaluation of the prediction uncertainty. Here the standard approach is to estimate the variance/covariance matrix of the regression parameters, based on the chosen error distribution model. This approach is, however, incapable of taking into account the propagation of reference material uncertainties.

This contribution described a calibration strategy which incorporates, on an equal footing, both the uncertainty of instrumental response and the uncertainty of the reference materials used as calibrants. The key elements of this calibration strategy are as follows:

(1) Optional choice among direct and inverse calibration.
(2) Arbitrary function types admitted.
(3) Regression calculation by the generalized least squares method (Deming, 1943).
(4) Independent validation by testing compatibility with the calibration data.
(5) Prediction uncertainty by error propagation, including correlations between reference material uncertainties.

This approach is currently used in several standardization projects in the fields of gas analysis, reference gases, and air quality measurement. The common objective of these projects is to implement the methodology of the recently published *Guide to the Expression of Uncertainty in Measurement* (ISO, 1993) in the calibration of analytical procedures.

### Confidence intervals for multivariate calibration based on neural networks and other non-linear methods

*Matthias Otto, University of Mining and Technology Institute for Analytical Chemistry, D-09596 Freiberg, Germany*

For validation of analytical methods, and for their quality control, confidence intervals are needed to judge the quality of the calibration parameters and the predictions of concentrations from the calibration model. With linear multivariate calibration methods the corresponding procedures are well established—at least for the K-matrix approach. Here the information can be derived from the variance-covariance matrix. However, for non-linear calibrations no agreed solutions exist. In the this paper the estimation of confidence limits by means of simulations, such as Boots trapping, was investigated and applied to different non-linear calibration methods. Among those methods are Neural Networks, NPLS (Nonlinear Partial Least Squares) and ACE (Alternating Conditional Expectations).

The principal steps for computing the confidence intervals and the statistical interpretations outlined. Practical applications were demonstrated for spectroscopic calibration in the NIR and mid IR-range, as well as for calibration of multiple channel sensors. Comparison of predictions and their related confidence intervals was also made with linear calibration methods, for example principal component regression.

### Quantitative analysis of ion mobility spectra using chemometric data expansion

*Lijuan Hu, Peter de B. Harrington, Ohio University, Center for Intelligent Chemical Instrumentation, Department of Chemistry, Clippinger Laboratories, Athens, OH 45701-2979, USA; and Dennis M. Davis, U.S. Army Edgewood Research, Development and Engineering Center, ATTN:SCBRD-RTE/(Dr. Dennis M. Davis), Aberdeen Proving Ground, MD 21010-5432, USA*

Ion Mobility Spectrometry (IMS) is a powerful technique for the analysis of trace amounts of organic compounds in the gas phase. IMS instrumentation is ideally suited for *in situ* measurement due to its low cost, portability, and real time monitoring capability. The response time is less than 30 ms to acquire a spectrum. Its disadvantage is low resolution, which makes it difficult for the analyses of mixtures. One solution to this problem is to couple a pre-separating system such as gas chromatography. However, the fast response and real time monitoring advantages would be lost. Also, IMS has a limited linear range which results in difficulty for quantitative measurements.

With the advances of chemometrics, expansion instead of compression can be used to facilitate data analyses. Several methods have been studied to process IMS data. Among them, artificial neural networks (NN) and Fourier transform deconvolution have been studied. Deconvolution is a useful preprocessing method for IMS data because it improves resolution. However, variations in peak width, number of peaks and noise may prevent the selection of a global set of parameters that furnish reliable models. Instead, an IMS spectrum may be expanded into an array for which the x-axis corresponds to drift time, the y-axis corresponds to a preprocessing parameter, for example, impulse response function, and the z-axis corresponds to intensity. Chemometrics models can be devised from the expanded spectra, and the models can determine locally optimal preprocessing parameters. Quantitative and qualitative models for IMS data of different mixtures were evaluated in this paper.

### Automatic fourier transform deconvolution in quantitative analysis of ion mobility spectra

*Peter de B. Harrington, Peng Zheng, Ohio University Center for Intelligent Chemical Instrumentation, Clippinger Laboratories, Athens, OH 45701-2979, USA; and Dennis Davis, Army Edgewood Research Development and Engineering Center, ATTN.: SCBRD-RITE/(Dennis M. Davis), Aberdeen Proving Ground, MD 21010-5432, USA*

Quantitative determinations by ion mobility spectrometry (IMS) are difficult, because IMS has a limited linear range and low resolution. Factors that may affect instrument response are pressure, temperature, and humidity. Nonlinear calibration methods, such as neural networks, may be ideally suited for IMS. However, these methods do not furnish reliable models when features of importance are not sufficiently resolved. Fourier deconvolution may be used to resolve overlapping peaks, and enhance the quantitative information.

An automated Fourier transform deconvolution algorithm has been developed. The width of the transfer function is an important parameter for deconvolution. A method that automatically determines this width was presented. The frequency domain representation of the spectra is processed, so that the method becomes resolution independent. The Fourier coefficients furnish a compressed representation of the data. This approach speeds up the network training rate and furnishes more general models. In addition, automatic frequency domain filters are determined by modelling the noise statistics in the data. The deconvolution process was evaluated with IMS spectra acquired from solvents. Techniques used to evaluate the deconvolution method were principal component analysis, partial least squares regression, and temperature constrained neural networks.

### A new Windows-based program to assess decomposition of drugs

*Roger I. Allen, Alex Avdeef and John E. A. Comer, Sirius Analytical Instruments Ltd, Forest Row Business Park, Forest Row, East Sussex, RH18 5DW, UK*

The authors studied the decompositions of several drug substances using a new potentiometric titration approach. For example, diacetylmorphine (heroin) rapidly hydrolyzes in to a mixture of 6-acetylmorphine and acetic acid in basic solutions ($pH > 11$); morphine-6$\beta$-D-glucuronide, a highly-active metabolite of morphine can partially decompose into a mixture containing glucuronic acid and morphine; aspiring degrades into salicylic and acetic acids; the antibiotic ampicillin appears to degrade into several molecules in basic solution.

The parent drugs and the by-products all contain characteristic ionization constants ($pK_as$) which were measured to very high precision. Precise knowledge of these constants allowed a computerized analytical procedure based on constrained mass-balance regression analysis of concentration factors in titration data to be developed. The method allows the degree of decomposition to be predicted.

The computer program is Windows-based, and uses data generated with the Sinus PCA101 ($pK_a$ and drug-lipid partition coefficient) analyser. This powerful computerized procedure may be applied for the determination of decomposition products of a wide variety of drug substances, and will be useful in the prediction of drug behaviour during storage.

### A simple pattern recognition based computer program for application to data from virtually any analytical technique

*Andrew D. Sauter, Microcomputer Application Inc., 217 Garfield Dr. Henderson, NV 89014, USA*

The application of pattern recognition across analytical chemistry is boundless. However, the routine application of this simple, but powerful, technology has been hindered by the availability of a user friendly, commercially available tool that can be employed to COMPARE data to whatever end in a standard environment. In this presentation, we introduce and demonstrate a simple computer program (an Excell add-in) that can be used to employ pattern recognition in any laboratory for data from virtually any analytical technique.

In this presentation, it was shown that pattern recognition can be employed to formally compare analytical *sample results* for analysis by GC/MS, ICP, alpha spectrometry and other techniques. The authors demonstrated how data is entered into the program, and how data can be transformed using a point and click approach to enhance or squelch different features of various data set. Chromatograms, retention time weighted chromatograms and toxicity weighted chromatograms were compared to show how data transformations can be astutely applied to provide insight to real world analytical situations across measurement techniques. COMPARE was employed to get out-of-specification analytical data into federal court in an actual nine figure litigation. Finally the authors explained how a series of vectors (i.e. forward and reverse COMPARE match values appropriately transformed and weighted) can develop a trajectory (a series of vectors) that points to the goodness or the badness of analytical data for virtually any analytical measurement technique.

### Automated extraction of petroleum source rock

*Kevin P. Kelly and Nancy L. Schwartz, OI Analytical, Sample Preparation Products Group, 555 Vandiver Drive, Columbia, MO 65202, USA*

Traditional Soxhlet extraction of source rock for petroleum characterization yields good results but it is slow and labour intensive. Source rock is difficult to extract; alternative methods (based on single partition static extraction) have not worked as well. Supercritical fluid extraction (SFE), for example, has provided lower recoveries of some petroleum fractions such as the bitumens and asphaltenes as evidenced by gravimetric analysis.

The automated Soxhlet extraction has been applied to gravimetric determination of fats or oils, and analysis of trace organics in soil or sludge. Its operating mechanism resembles a traditional Soxhlet; however, during the first processing step sample is suspended in refluxing solvent, causing more rapid extraction. Following this, a second total reflux period occurs during which sample is suspended *above* boiling solvent, with the sample and thimble thoroughly rinsed by the condensate, thus providing complete and precise recoveries. The automated Soxhlet yields results equivalent to traditional extraction, but it is more rapid and generally uses less solvent.

Application of automated Soxhlet to extraction of source rock was investigated using an automated system. All stages of operation were unattended, resulting in lower labour requirements. Dried rock samples (powdered or crushed) up to 75 g were processed using $\Leftarrow$ 135 ml of methylene chloride. Extracts were automatically concentrated to small volume (evaporation was halted at a concentrate volume of 10 to 20 ml to avoid overheating of the extract, which might alter some of the petroleum components). The distillate produced during the evaporation was recovered in an internal tank, facilitating solvent disposal or recycling. When necessary, extract evaporation was completed at room temperature using a nitrogen stream.

Results were presented for extraction of materials such as seep samples, sandstone, core plugs, and coal. Gravimetric recoveries and analytical profiles were compared between extracts of the same material produced using either traditional or automated Soxhlet extraction.

### Automatic capillary GC: a powerful tool for petroleum products characterization

*J. P. Durand, Institut Francais du Pétrole, BP 311, 92506 Rueil-Malmaison, France and L. Joubert, Vinci Technologies, BP91, 92503 Rueil-Malmaison Cedex, France*

For characterization of petroleum products, spectroscopic techniques using infra-red have the advantage of being much faster than GC. Capillary GC gives more chemical information and, as the separation depends on the boiling point of each compound, a simulation of distillation can be carried out on the crude sample. The high resolution of capillary columns allow several hundreds compounds up to C20 hydrocarbons to be separated. With the advances in the reliability of chromatographs and columns, using a data system, the development of automatic capillary analysers has become a possibility.

A software named Carburane, developed by IFP and marketed by Vinci Technologies (France), carries out the chromatographic data handling to automatically identify the chromatogram. It has been updated according to the many opportunities given by a detailed analysis, specifically physical properties determination and simulation of distillation. Carburane works in post-run of the chromatographic PC station (Hewlett Packard and Varian) and can be adapted to any other station. Chromatograms are identified by a reproducible index system which requires an adjustment of chromatographic conditions. The software controls the adjustment of this parameters. Data handling after identification consists in resolving the possible interferences, in classifing the compounds by hydrocarbon group types, carbon numbers, fractions of distillation, in giving the results in weight %, mole %, volume % and in determining the elementary analysis and different physical properties (specific gravity, molecular weight, vapour pressure, heat value, octane number). With this software, numerous automatic chromatographic methods and an industrial on-line analyser has been developed.

Carburane is used in geochemistry (oil migration, source rock extract), for reservoir fluids studies (molecular weight distribution up to C20), in refining (analysis of gasoline pool, monitoring plant performances), to characterize the fuels (detailed analysis and calculation of physical properties on motor gasolines), and to control soil pollution by fuels (soil extract).

The on-line process analyser is computer controlled and the laboratory equipment is installed in an explosion proof shelter. It automatically performs the detailed analysis of different effluents and determines their physical properties.

### The analysis of PPB metal hydride impurities in specialty gases by GC/RGD

*William M. Geiger and Carol J. Meyer, Merlin MicroScience, Inc., P.O. Box 19528, Houston, TX 77224, USA*

The analysis of metal hydride impurities in specialty gases is of concern to propylene and polypropylene manufacturers. These impurities can cause deleterious effects to catalysts used in processing propylene in polypropylene manufacturing processes when present even at low ppb concentrations. The Reduction Gas Detector has been found to have ppb sensitivity to these hydride impurities and has been used in conjunction with a gas chromatographic system to separate and quantitate phosphine, and arsine impurities in high purity propylene.

The mechanism of detection of the RGD is the selective reaction of reducing gases with a mercuric oxide bed followed by the detection of the freed mercury by means UV absorption. Because of the strong absorption of mercury, this detector is extremely sensitive. However, due to the reaction chemistry of the detection scheme, complex interactions can occur between components which are not separated prior to the reaction in the mercuric oxide bed. In addition to hydrides, the detector is sensitive to other reducing agents which occur in this analysis including propylene, acetylene, ethylene and carbonly sulfide. While several of these are of interest to propylene manufactures, they occur at levels which may be problematical in the analysis of extremely low levels of metal hydrides. Therefore the separation scheme becomes important to the use of this detector.

A chromatographic scheme has been developed which is capable of the analysis of ppb level phosphine, arsine and carbonyl sulphide in propylene. This scheme has been incorporated into a process platform and is capable of repeated analyses of propylene without significant artifacts.

### *In situ* infra-red monitoring of emulsion polymerizations

*Alan J. Rein, Steven M. Donahue, ASI Applied Systems, 8223 Colverleaf Drive, Suite 120, Millersville, MD 21108, USA; and E. David Sudol, Emulsion Polymers Institute, Lehigh University, Iacocca Hall, 111 Research Drive, Bethlehem, PA 18015, USA*

Manufacturers continue to develop new and ever more complicated polymerization process. New tools and techniques are required to help characterize these processes so that products can be brought to the market quickly while being made safely and efficiently. This is particularly true for emulsion polymerizations. Even in their simplest form, these processes involve very complex chemistry. To date most studies of emulsion polymerizations have involved removing a sample from a reaction mixture and subjecting it to a variety of analytical tests. There is always the question of whether the act of sampling alters the sample in some way. The problem is further compounded when sampling non-homogeneous reaction mixtures.

*In-situ* techniques have proven invaluable in these situations. ASI Applied Systems ReactIR reaction analysis system has been used to characterize several emulsion polymerizations. Using an insertion probe with an internal reflection sensor monomer uptake and extent of reaction are easily monitored.

This information alone would be of value to process engineers. However, *in-situ* infra-red may prove more useful for characterizing the physical properties of the emulsion. In studies of emulsions, miniemulsions and microemulsions, the absorbance profiles were found to be dependent on the droplet size of the active species of the reaction. Infra-red data have been correlated with particle size and calorimetric measurements. Results were presented for two reactions; the polymerization of butyl acrylate and the copolymerization of vinyl 2-ethylhexanoate and vinyl acetate.

### In-line monitoring of composition and colour in polymer extrusion

*Stephen T. Balke, Ramin Reshadat, Sina Sayed, Saed Sayed, William R. Cluett, University of Toronto, Toronto, Ontario M5S 1A4, Canada; Felix Calidonio, Christopher J. B. Dobbin, Colortech Inc., Brampton, Ontario, Canada, and Jeffrey W. Hall, NIRSystems, Inc., Silver Spring, MD, USA*

A fibre optic assisted near-infra-red spectrophotometer was used to monitor colour and composition in polyolefin melts during extrusion. Both diffuse reflectance and transmittance monitoring at different points in the extrusion process was employed. In-line colour measurements were accomplished by using wavelengths from 400 to 780 nm in the absorbance spectrum while composition measurements utilized 1100 to 2500 nm. Colour concentrates contained 10 to 60 wt.% inorganic pigments in Linear Low Density Polyethylene (LLDPE). A comparison of in-line and off-line colour monitoring of these concentrates showed that off-specification product could readily be distinguished from product which was within specification. Temperature effects on pigment colour were also observed. Methods of predicting colour of the final cooled product from the absorbance spectrum of the polymer melt were investigated. Excellent predictions were obtained using artificial neural network software. In-line composition measurements of polyethylene-polypropylene blends were interpreted using several multi-variate methods. Best results were obtained using partial least squares with the percent polypropylene predicted to within approximately 0·5 wt.%. Currently, measurement of colour and composition simultaneously in the same extruder run is being investigated.

### Monitoring gas-phase production processes with near-infra-red spectroscopy

*Rick L. Cox and Peter J. Brush, Perstorp Analytical, Inc., 12101 Tech Road, Silver Spring, MD 20904, USA*

Near-infra-red (NIR) spectroscopy has been routinely used for the analysis of strongly absorbing species such CN, NH and OH entities in condensed phases (i.e. liquids, solids). However, the improvements in instrumentation, fibre optics and sampling interfaces affords the opportunity to analyse gas-phase reactions with NIR spectroscopy.

By use of fibre optics and a long optical pathlength gas cell, direct analysis of acetylene during an ether production process was monitored. This presentation provided a practical description of the steps necessary to implement NIR spectroscopy for monitoring gas-phase production processes.

### On-line analysis of environmentally important parameters in the refining industry using NIR

*Larry McDermott, The Perkin Elmer Corporation, 50 Danbury Road, Wilton, CT 06897, USA*

Motor vehicle emissions are a major contributor to worldwide air pollution. Governments are regulating changes in fuels including gasoline and diesel fuel in an attempt to decrease air pollution. While the technical and political merit of the changes will be debated into the next century, the changes required have already begun to take place with the introduction of reformulated fuels. Fuels will be regulated on an increasing number of both chemical and physical parameters.

Parameters to be measured using upcoming fuel models include: percentage aromatics, percentage olefins, 50% distillation point, E200 and E300, benzene, reid vapour pressure and oxygenates. Many of the parameters regulated can be analysed simultaneously, along with key consumer performance parameters such as the octane number of gasolines and the cetane number of diesel fuels. Refiners can measure these values on-line as they are blending their final product, or upstream at the individual process units, and can adjust their processes to keep the finished product within specification.

This paper reviewed the performance of measurements made on these properties attained with an on-line process near infra-red analyser, and compared these results with performance specifications attainable in the laboratory using official methods of analysis.

### Educational applications of computer based environmental modelling

*James M. Ferguson and H. M. 'Skip' Kingston, Environmental Science and Management Program, Duquesne University, Pittsburgh, PA 15282-1503, USA*

Models have been widely used in environmental science since the late 1960s when the discussion of pollution became prominent worldwide. The complexity of the environment often necessitates the use of sophisticated environmental models and the use of such models, particularly computer models, has become an essential part of managing the impact of human advancement and technology on the environment. With ever-increasing emphasis on modelling as part of the process of prediction, planning, and policy, it is apparent that modelling needs to become a requisite part of any educational environmental programme. Experience with the Environmental Chemistry course within the Graduate Environmental Science and Management programme at Duquesne University has proven the benefits of integrating professional computer modelling tools into the classroom and laboratory.

Because models must simplify reality, they are subjective. Hence, the modelling process itself may provide a stimulus for thought since it requires a close examination of the relevant features and relationships of the system being studied. A model using the spreadsheet program Excel was created for this very purpose — to use a model to describe, explore, and analyse how a system works. Furthermore, a co-operative learning project and student laboratory assignments on an established metal speciation model (MINTEQA2) expanded classroom discussions and examined the assumptions and limitations built into computer modelling. A discussion of how to obtain free US Government models and utilize them in teaching environmental chemical concepts at multiple educational levels was presented.

### An electronic nose for food and meat control

*Laurent May, Alpha MOS-SA, 3, avenue Didier Daurat, 31400 Toulouse, France*

Among the first of the applications of the electronic nose technology was the food industry. Many leading industrial companies are now equipped to use this technique to identify, qualify and manage odour and volatile compounds problems.

The nose has been used in application fields such as spices and food aromas, chocolate, coffee, butter, beers, fish chewing gum, cereals and grains.

In this presentation, discrimination and identification of different meat quality and meant taint from various animals and according to several techniques of preparation (smoked, dried and grilled) was explained.

Several statistical techniques have been tested to analyse the data and build reliable models to keep the validity of a meat data bank. The models include the use of classical chemometrics, as well as neural network and a fuzzy logic.

### QC methods using infra-red spectroscopy: hardening a laboratory method for QC application

*Dean E. Roberts, Mattson Instruments 1001 Fourier Dr., Madison, WI 53717, USA*

Successful transfer of an analytical method to the QC lab requires more than just including the conversion of spectral data to a number or pass/fail result. Often, regulatory agencies require proof of method validity and on-going checks of the method's precision and accuracy. In addition, samples that fail QC tests may fail for reasons other than the sample itself: sample preparation, measurement conditions, and interferences are among the more common causes. Taking account of the possible causes of error in the QC analysis, and building check routines into the method hardens and method and helps eliminate costly errors in quality control measurements. Auditing the validity of spectral data and results from within a QC method was discussed in detail in this paper. In addition, methods of assessing method precision and accuracy were proposed.

### Remote detection of environmental pesticide spray using FTIR spectroscopy

*Jack C. Demirgian, Gregory F. Busse, Analytical Chemistry Laboratory, Chemical Technology Division, Argonne National Laboratory, 9700 South Cass Avenue, Argonne, IL 60439-4835, USA; and John Ditillo, U.S. Army Edgewood Research, Development, and Engineering Center, ATTN: SCBRD-RTE, Bldg. E5554, Aberdeen Proving Ground, MD 21010-5423, USA*

Pesticides are commonly used to spray garden and ornamental plants. The extent to which this spray spreads depends on wind speed and direction. When large amounts of spray are released for agricultural purposes, the amount that could spread beyond the intended area may be quite large. The ability to detect pesticide spray by using remote instrumentation would provide security that the spray remains in the intended area and would also provide a means of identification of which pesticide was sprayed.

The objective was to determine the ability of a commercially available (Midac) emission spectrometer to detect malathion spray in the environment and to differentiate it from compounds that have similar spectral absorbances. A specially designed vaporizer was constructed to release exact quantities of malathion and other chemicals with similar spectral absorptions. Data were collected using grass, low sky, and mid-sky backgrounds. Detection levels were at the low ppm-m level.

The data were analysed under different release conditions and backgrounds. A major spectral interference is atmospheric ozone, especially prevalent during the summer. The ability of different algorithms to differentiate between malathion and interfering compounds such as atmospheric ozone were discussed.

### Simultaneous one minute field analysis of hydrogen, helium, methane, ethane and carbon dioxide in soil gas samples

*K. R. Carney, G. Hunt, N. J. Roques, and E. B. Overton, Institute for Environmental Studies, 42 Atkinson Hall, Louisiana State University, Baton Rouge, LA 70803, USA*

Field analysis of environmental samples generally involves detection of analytes with volatilities like those of vinyl chloride and dichlorobenzene. However, there are many important applications that require analysis of analytes like hydrogen and helium as well as methane, ethane and carbon dioxide. For example, elevated concentrations of hydrogen, helium and methane in near surface soil gas have been variously associated with geological phenomena such as deep oil reservoirs, mineral deposits, fault structures and impending fault movement. Additionally, the generation of small quantities of hydrogen during the oxidation of iron under low oxygen conditions offers a technique for monitoring corrosion of underground storgage tanks and pipelines. Methane and hydrogen concentrations can provide information on the redox state of the soil and anomalous values may indicate the presence of organic contamination.

The high volatility and, for hydrogen, reactivity of these compounds makes returning the large number of competent samples required for surveys of even moderate areas extremely difficult. The authors have developed a method for rapidly sampling and analyzing near surface soil gas samples with a dual column micro gas chromatograph.

The method requires about 5 minutes to collect and analyse a sample from a depth of 5 to 6 feet. Because only a small volume ($<2$ ml) of sample is required, samples can be simultaneously collected for transport to the laboratory as desired. Analysis can be performed on-site in less than one minute for hydrogen, helium, methane, ethane and carbon dioxide at concentrations down to 1 ppmv using a 10 m MolSieve 5 Å column in parallel with a 10 m PoraPlot Q column. A team of three can analyse about 75 to 100 samples per day.

The authors have used this method in the context of oil exploration in southern Mississippi, profiling a transect across a fault zone in Baton Rouge, LA and investigating subsurface geological features of a salt dome in south Louisiana.

### A microprocessor controlled sample processing system for field voc analytical instruments

*E. B. Overton, H. P. Dharmasena, and K. R. Carney, Institute for Environmental Studies, 42 Atkinson Hall, Louisiana State University, Baton Rouge, LA 70803, USA*

A number of analytical devices are currently available for accurate and precise analysis of complex samples in the field. Virtually all of these instruments are designed only for analysis of compounds having vapour pressures above about 0·5 mm Hg. Further, these instruments are designed only for analysis of gas phase samples, limiting their application to air or static headspace analyses.

The authors have developed a flexible, automatic sample processing module that expands the applicability of various field analytical instruments to include dynamic headspace analysis of waters and soils and also lowers detection limits for air analysis by as much as three orders of magnitude. The sample processor uses two stage sorbent concentration and requires less than 25 W of power during continuous operation. The unit can be added on to most instruments using either sample loop or on-column injection. The concentrator can also be bypassed simply by sending a command from a host computer. The authors used this sample processor with a micro machined gas chromatograph to lower detection limits by more than two orders of magnitude.

The microprocessor controlling all functions of the sample processing unit automatically synchronizes the cycle of the sample processor with that of the analytical instrument. The sample processor can share a serial port with the analytical instrument if necessary. Once installed, the sample processor module is totally transparent to the control and acquisition system used by the analytical instrument; no modifications of the original software is required to use the sample processor module.

### Application of a mobile GC-MS-system in case of fires and chemical accidents

*Gerhard Matz, Alexander Harder, Alfred Schillings, Technical University Hamburg-Harburg, Department of Measurement Technology, Harburger Schloss-Str. 20, 21071 Hamburg, Germany; and Peer Rechenbach, Fire Department of Hamburg, Westphalensweg 1, 20099 Hamburg, Germany*

A new mobile GC-MS-system was used for fast analysis of hazardous organics in fires and chemical accidents. Fast identification and correct assessment on-site of possible hazard for the population and the environment is essential. GC-MS was recommended as the best technique for identification and quantification of most organic compounds especially in complex mixtures. The new mobile GC-MS-system EM640 (Bruker) was used because it is a compact, mobile and rugged instrument. This GC-MS-system has been developed into a semi automatic machine, which can be used by fire-fighters.

Sampling equipment for air, water and soil are used by 20 German professional fire-brigades. Since 1992 they have taken samples at 150 real actions (fires, chemical spills, chemical accidents). Samples are sent to the Technical University of Hamburg-Harburg and analysed by mobile GC-MS-system. The GC-MS-system has been tested on site at real actions in co-operation with the fire-brigade.

Separation on short columns decreases analysis time to 5 min. Over 300 different organic compounds have been identified by MS. The results are integrated in a database for further evaluation. Special MS-databases have been built which operate hierarchical for fast and automatic identification with high reliability.

This paper described how a complex analysis (sampling, analysis by GC-MS and assessment) can be done in a few minutes by non-specialist staff.

### Portable X-ray fluorescence for the determination of heavy metal contamination in soil on firing ranges

*John F. Schneider, Lorraine M. Hahn, Jae K. Lee, Argonne National Laboratory, 9700 South Cass Avenue, Argonne, IL 60439, and Albert Bohm, Environmental Management Office, 282D BSB Hohenfels, DEH, Germany*

The objective of this investigation was to identify the types and degree of heavy metal contamination on selected firing ranges at the Combat Manoevre Training Center (CMTC), Hohenfels, Germany. A field investigation was conducted, using portable X-ray fluorescence (XRF) to detect heavy metals directly in the soil. The value of portable XRF as a screening tool for lead and other heavy metals in soil is generally accepted. As part of the study, the readings obtained with the XRF were compared with values obtained from soil samples analysed by atomic absorption spectrometry (AA). Certified standard reference material (SRM) soils were used. Results of the comparison with AA and the utility of portable XRF for the determination of heavy metal contamination in soil on firing ranges were discussed.

### On-line chromatographic ICP-MS analysis of traditionally difficult environmental samples

*Dan Taylor, Don Nogay, Dengwei Huo and H. M. 'Skip' Kingston, Department of Chemistry and Biochemistry, Duquesne University, Pittsburgh, PA 15282-1503, USA*

On-line sample manipulation methods can be used to improve the accuracy and precision of measurements, lower limits of detection, and provide more information about environmental samples. Researchers need to determine not only the total concentration of a metal in a sample, but also the form, or speciation of the metal to evaluate its toxicity and fate. The speciation of chromium is of particular interest, because of its wide use in industry. Chromium exists primarily in two forms. Cr(III) is a nutrient needed in trace amounts by mammals, and Cr(VI), a strong oxidant, is a known carcinogen. A technique for determining the speciation of chromium in environmental samples was described. The chromium species are separated by ion exchange chromatography using an anion exchange column. The concentrations of the species are then determined by on-line isotope dilution using an ICP-MS. The technique is used to determine the chromium species in environmental water and soil samples.

Improved accuracy and precision in complex matrices can be achieved with solid phase chelation. On-line solid phase chelation has been used to determine the concentration of Cd, Co, Cu, Mn, Ni, Pb, U, and Zn in the certified reference materials CASS-2, near shore seawater, NASS-4, open ocean seawater, and 1643b, trace elements in water. The method uses a column packed with an iminodiacetate resin and a commercially available low pressure sample manipulation system to preconcentrate analytes and eliminate the matrix elements. The detection limits for 10 ml samples ranged from 0·8 ng $l^{-1}$ for Co to 40 ng $l^{-1}$ for Cu and Zn.

### Rapid semi-automated characterization of headspace VOCs in transuranic waste containers by at-line FTIR

*W. F. Bauer, Idaho National Engineering Laboratory, Lockheed Martin Idaho Technologies, Idaho Falls, ID 83415, USA; D. Gravel, A. Rilling, H. Buijs and S. Perry, Bomem Hartmann & Braun, Québec, Québec, Canada G2E5S5*

Conventional analysis of headspace VOCs in transuranic waste containers is performed with SUMMA canister sampling followed by GC analysis with either thermal conductivity, flame ionization or mass spectrometric detection. These analysis methods can be both time consuming and costly. To reduce turnaround time and cost, the authors have developed an automated at-line analysis system which transfers headspace gases directly into an absorption cell of an FTIR. The FTIR has been calibrated to automatically quantitate 29 VOCs and methane in the presence of a number of common interferents such as water vapour, carbon dioxide, nitrous oxide, trimethylamine, ammonia, and $> C_6$ hydrocarbons.

The performance of the at-line FTIR-based VOC analysis system was evaluated by direct comparison to GC analyses of actual waste drum headspace and by daily evaluation of standards. Long term precision was found to be $< 10\%$ and typically $\sim 5\%$. Accuracy was well within the $\pm 30\%$ required for the program as it usually fell in the 5–15% range.

### Quantitative analysis of volatile organic compounds by passive FTIR remote sensing measurements

*Ndumiso A. Cingo and Gary W. Small, Center for Intelligent Chemical Instrumentation, Department of Chemistry, Clippinger Laboratories, Ohio University, Athens, OH 45701, USA*

The development of chemical sensors for determining hazardous organic compounds is one of the areas of environmental monitoring generating widespread interest. Recent work in the authors' laboratory has focused on the development of data analysis methodology for use with sensors based on Fourier transform infra-red (FTIR) spectroscopy that can be used for remote monitoring of smoke stack emissions. Quantitative analysis of smokestack emissions by remote-sensing measurements is complicated by lack of proper reference spectra for use in obtaining absorbance spectra of the analyte, lack of a stable infra-red background, occurrence of overlapping spectral bands due to other IR-active species present in the field of view of the spectrometer, variation in the path length of the absorbing species, and the need for a low cost, rugged, and reliable spectrometer able to withstand field measurement requirements. To address these problems, work performed in the authors' laboratory has focused on the design and application of digital bandpass filters directly to short interferogram segments of FTIR interferograms in order to isolate spectral information specific to the analyte of interest. The effect of digital filter design techniques on the quantitative analysis of remote sensing data of volatile organic compounds such as trichloroethylene, acetone, methyl ethyl ketone, sulphur dioxide and methyl chloride were evaluated. Both laboratory and field remote sensing data were used in this work.

### Automated GC peak analysis using a hybrid neutral network

*Taiwei Lu, David Mintzer, Sean Zhao, Igor Tebelev, Physical Optics Corporation, 20600 Gramercy Place, Torrance, CA 90501-1821, USA; Mel Grundleger and Jan Hazebroek, Pioneer Hi-Bred International, Incorporated, Johnston, 50131-1004, USA*

Soybean, sunflower and canola seeds are bred for qualities such as taste, geographical suitability, protein and oil content, and pest and disease resistance. The oils of these seeds contain many classes of compounds. Gas chromatography (GC) is commonly used to identify the components, with most of the GC data being examined by expert technicans. A customized hybrid neural net (HNN) has been developed to perform automated analysis of gas chromatograms of seed oils. The HNN is capable of performing on-line chromatographic analysis—analysing chromatograms of multiple compounds with many features in real time.

The HNN GC data analysis system works in three stages: (1) feature extraction; (2) pattern recognition; (3) and decision making. The feature extraction stage utilizes conventional signal processing techniques such as correlation, fast Fourier transform, and partial least squares to reduce a set of 450 raw data points to 20 characteristic parameters. A three-layer feedforward neural network with 40 input neurons is used to classify chromatogram features with extremely truncated peaks, interfering peaks, and electronic spikes. The HNN accomodates retention time offsets, vaccenic acids, instrument faults, and weak signals. In the decision making stage, multi-level thresholding values are chosen by the user so that the results from the HNN are operator independant.

The GC classification system was trained within 10 minutes on a 486-based PC, equivalent to 2000 epochs, and approached 100% accuracy over 400 test case chromatograms with an analysis time of 20 ms per sample.

The entire system was designed as a Windows 3·1x callable DLL.

### Multi-system HPLC control software based on Microsoft Windows NT

*Rick Gard and André Szczesniewski, Hitachi Instruments, Incorporated, 3100 N. First Street, San Jose, CA 95134, USA*

With the advent of modern 32-bit multitasking operating systems, such as Microsoft Windows NT and Windows 95, the 16-bit desktop operating system has finally passed into history. This presentation introduced new HPLC instrument control software based on the leading-edge Windows NT operating system. This operating system offers the US Government's C2-level security certification, and high fault tolerance to support mission critical operation, in addition to several other advanced services designed to support even the most demanding technical applications. Because of new operating systems, such as Windows NT, desktop computers are now more powerful than ever, and provide a host of extended features that go beyond just the convenience of a standard graphical user interface.

Other modern features of this new software include a CD-ROM based multimedia maintenance and help system, as well as password protected file security and audit trail tracking of data files. Features such as built-in networking and dynamic data exchange (DDE) with spreadsheet and word processing applications, allow complete flexibility in reporting and postrun processing of data. Instrument confidence log books are automatically maintained on each hardware component, keeping track of the use and condition of each consumable part such as lamp hours, lamp energy, wavelength accuracy, and pump or autosampler seal wear.

### Managing instruments and tasks in LIMS

*Dr Jos. Webber, LabWare Inc., 3 Mill Rd, Suite 102, Wilmington, DE 19806, USA*

The majority of modern pharmaceutical laboratories use a Laboratory Information Management System (LIMS) to help manage sample information and result data. Some laboratories also have integrated instruments connected to their LIMS applications. However, as the need for improved efficiency and data quality in the laboratory increases, laboratory managers are now looking for the ability to schedule and manage their workloads from one central integrated system.

Many LIMS systems are completely disconnected with the scheduling and management of work within the laboratory, work can be assigned to instruments that are off-line or out of calibration, and scheduling of work to analysts is often overlooked. To correct for these deficiencies, some laboratories elect to modify a commercial LIMS application to add in work management features. This in turn leads to issues with system validation and upgradability.

This paper looked at the design of a Client Server LIMS system integrated with an instrument management system. The system provides instrument and task management in addition to the more traditional LIMS features like sample login and test assignment. Working examples of laboratories using these systems will be provided. The management and control of immunoassay tests and protocol based studies was also be addressed. The current status of work management within the laboratory was reviewed and future directions were discussed.

### Noninvasive clinical chemistry with near infra-red spectroscopy

*Mark A. Arnold, Department of Chemistry, University of Iowa, Iowa City, IO 52242, USA*

The prospects of performing noninvasive clinical chemistry measurements with near infra-red (NIR) spectroscopy are intriguing. Conceptually, such measurements involve passing a selected band of NIR radiation through a region of the body, collecting the transmitted/scattered radiation and then extracting concentration values for selected analytes from the resulting spectral information. Pulse oximetry is an excellent example of this approach where oxygen saturation is measured based on the absorption characteristics of deoxyhaemoglobin. The significance of this technology stems from its ability to provide *in vivo* physiological information in a continuous and real-time manner.

Can this approach be used for other clinically relevant analytes? Noninvasive blood glucose measurements are of particular interest, given the need for individuals with diabetic mellitus to maintain tight control of their blood glucose levels. Continuous blood glucose measurements from a noninvasive device would provide the information needed for tight control and disease management.

A primary question to be addressed is the feasibility of measuring glucose noninvasively by NIR spectroscopy. The author's aim was to establish the ability of NIR spectroscopy to measure glucose in biological matrices of increasing complexity. The author started with glucose in a simple phosphate buffer and progressed to the analysis of whole blood matrices. Valid calibration models have been generated with each matrix. Successful models require spectra with high signal-to-noise ratios coupled with partial least squares (PLS) regression analysis.

The next step was to assess the feasibility of extracting blood glucose information from NIR spectra collected in a noninvasive manner from human subjects. An experimental model of the noninvasive human subject experiment has been developed to aid in our basic understanding of these difficult spectroscopic measurements. This model allows the critical experimental parameters that influence noninvasive blood glucose measurements to be identified and evaluated.

This presentation summarized progress in developing and assessing methods for measuring *in vivo* blood glucose levels with NIR spectroscopy. In addition, results reported by others claiming success in generating valid blood glucose models from NIR spectra collected noninvasively were critically evaluated.

### ICP-AES for process monitoring: an instrument choice worth considering

*Gerhard A. Meyer, Robert Foster, C. Carroll, and T. Toothaker, Thermo Jarrell Ash, 27 Forge Parkway, Franklin, MA 02038, USA*

Federal and state legislation regulating municipal and industrial effiuents has brought renewed interest in applying established laboratory chemical analysis methods to real-time monitoring and control. Demand for more frequent sampling and decreased analysis turnaround time has prompted the resurgence of more technically sophisticated process analysers to the marketplace. The reduction of toxic chemicals emission into the environment through tougher federal mandates is also creating an increased awareness throughout industry that better and more economical chemical instrumentation is needed to maintain profitability within the confines of the law.

Elevated manufacturing efficiency, regulatory compliance assurance, and industrial hygiene maintenance are some of the biggest advantages to real time continuous monitoring. Of the available atomic sepectrometries for determining metals, the ICP combines the widest range in elemental linear dynamic range and sensitivity at a moderate cost. Over the last several decades, ICP-AES has become widely accepted as a fast and reliable method for metals analysis. Together with proven long term stability and economical cost of operating on a continuous basis, the ICP has emerged as a strong candidate for real time monitoring of metals in a variety of liquid process streams. This presentation reviewed features of the ICP that make it the instrument of choice for performing real time metals determination. Several examples of where ICPs have been successfully solving industrial monitoring problems were given.

### Determination of mercury at the PPT level

*Doug Shrader, Varian Optical Spectroscopy Instruments, 201 Hansen Court, Suite 108, Wood Dale, IL 60191, USA; and Brian Frary, Varian Australia, Pty Ltd, 679 Springvale Road, Mulgrave 3170, Victoria, Australia*

Mercury is a commonly occurring element and is present almost universally in the environment due to its use by man over the centuries. Mercury vapour and its soluble salts are highly toxic. Due to its toxicity, the determination of mercury is required in biological samples, food, water, waste and other environmental samples. Government regulations prescribe the allowable limits in various matrices. Regulations may require mercury to be determined at levels as low as 10 ppt (ng/l).

Determination at these low levels raises problems not evident at higher levels including:

(1) Blank level of reagents.
(2) Contamination of apparatus used in the collection and storage of samples.
(3) Contamination during sample processing and measurement stages.
(4) Sensitivity and stability of the mercury measuring device.

These problems were discussed and solutions proposed. Solutions presented included a new mercury measuring device (i.e. dedicated mercury analyser). Results for the determination of mercury in various samples (including reference materials) were presented along with quality control data. Results showed that the accurate determination of mercury at $<10$ ng/l is routinely possible.

### Monitoring of emulsion polymerization using Fibre-optic Raman spectroscopy

*A. Al-Khanbashi, M. G. Hansen, Department of Chemical Engineering, 419 Dougherty Engineering, University of Tennessee, Knoxville, TN 37996, USA; and E. A. Wachter, Health Science Research Division, Oak Ridge National Laboratory, Oak Ridge, TN 37831, USA*

Raman spectroscopy is a powerful and versatile tool for the study of a wide variety of materials. Recent advances in fibre-optic Raman have attracted great attention in developing methods for monitoring chemical processes in remote and hostile enviroments. In polymer production, emulsion polymerization is a major commercial process for polymerization of various polymers. This work described *in situ* monitoring of the emulsion polymerization of vinyl acetate using remote Raman spectroscopy. A dispersive spectrometer fitted with a charge-coupled device detector was used with excitation from a low power helium neon laser. Laser excitation was introduced through a single fibre, and the scattered light is collected using additional optical fibres.

The disappearance of vinyl acetate was followed by monitoring the vinyl carbon stretch band at approximately 1650 $cm^{-1}$. Multivariate methods involving the use of singular value decomposition to perform principle component regression and partial least squares regression were used to develop a calibration model that quantitatively describes the change in the monomer concentration which can be related to the reaction rate.

### Automated SFE-HPLC via off-line liquid trapping for the analysis of high extractable-content samples

*Phillip B. Liescheski and Dale L. Clay, Isco, Inc., Separation Instruments Division, 4700 Superior, Lincoln, NE 68504, USA*

Supercritical Fluid Extraction (SFE) continues to replace traditional sample preparation methods used in QC analyses. With automated SFE, the cost and time involved in sample preparation are reduced; however, the transfer of the extract to the analytical assay instrument is still typically a manual process. This paper described a method for automating the entire extraction and analysis via off-line liquid trapping coupled directly to HPLC. After collecting the extracted analyte in the liquid trap, the SFE system automatically and volumetrically adds an internal standard and dilutes the extract. An additional liquid pump can automatically deliver an aliquot of the extract to the injection loop of an HPLC. This allows the users to automate the extraction and HPLC analysis of solid samples.

On-line SFE-chromatographic coupling techniques generally load all of the extracted analyte onto the analytical column. This allows for very high sensitivity, which is desirable for environmental samples with analtyes at the sub-ppb level. For food or polymer samples, however, analyte concentrations are typically in the high ppm or even percentage range. On-line coupling in these applications can lead to column overload or unrealistically small samples sizes. The off-line coupling technique described here avoids this limitation by loading only a small, controlled fraction of the extracted analyte onto the column.

This paper presented work done on spiked polymer additive samples. The coupling technique and instrumentation were briefly discussed. The precision of the internal standard addition and the HPLC analysis as coupled to SFE was investigated.

### Routine, automated sample preparation using SFE for the extraction of additives from polyethylene and polypropylene

*Anita Cardamone and Athos C. Rosselli, Suprex Corporation, 125 William Pitt Way, Pittsburgh, PA 15238, USA*

This paper described the application of a novel automated supercritical fluid extraction system for the routine determination of additives in polyolefins in both plant process and technical service laboratories. Data illustrating the advantages of SFE over conventional methods was shown in a QC setting, as well as measures of the productivity gains obtainable with an automated system. The system itself was described, and the methods optimization process discussed. Repeatability and recovery data for the extraction of additives from polyolefins were presented. The effect of pre-extraction sample preparation and modifier addition and their effect on extraction kinetics, recovery and extraction duration was also discussed.

### Electrochemical immunosensors and monitoring environmental pollutants

*Omowunmi A. Sadik and Jeanette M. van Emon, US-EPA/National Exposure Research Laboratory, Characterization Research Division, PO Box 93478, Las Vegas, NV, USA*

A practical immunosensor for environmental monitoring should be specific, reversible, able to provide fast response time, and capable of direct detection of an immunoreaction, with minimal sequential addition of immunoreagents. Also, the sensor should be capable of continuous flow measurements and have capabilities to determine multiple analytes in complex samples, with no need for sample preparation steps. Finally, the immunosensor system should be able to process signals, and be suitable for integration into other devices that can excercise real-time feedback as required for pollution monitoring. Although a number of sensing methods for the measurement of pollutants have been reported, very few address these specific requirements.

The combination of electrochemical immunosensors with flow injection analysis offers great advantages in the development of readily automated, high sample throughput, compact, and moderately inexpensive instrumentation that can satisfy (or are potentially able to satisfy) the above-mentioned criteria. The United States Environmental Protection Agency, Las Vegas, is carrying out research in the development of immunosensors to study the release of pollutants into the environment for human exposure studies. The system involves the use of an electrochemical transduction principle for measurements in liquids. The sensor consists of a portable, low-cost, electronic module which sends an electrical potential wave across a flowing sample, and a reference electrode that is sensitive to potential changes at the surface of an electronically responsive polymer membrane. Reversible analyte recognition is achieved by using antibody-protein molecules having specificity and affinity for the target analytes. The system can be used as an alarm station for early detection and full-time surveillance of pollution in groundwater, waste stream effluents, agricultural run-offs and for monitoring the effectiveness of remediation.

The reversible control of the antibody-antigen interaction is particularly crucial to the development of a renewable sensor surface. First, a large number of important biological and environmental molecules can be detected in real time, as opposed to the use of a complex biochemical procedure. Secondly, there is no net degradation of the sample. As the assays are produced electrochemically, there is also a clear path to their implementation on interdigitated microelectrode arrays. This yields the advantages of improved sensitivity, multi-analyte sensing, and the possibility of integration into other devices for convenient signal processing. This paper described an overview of electroanalytical techniques employed for immunological detection, and discussed the development of an on-line immunosensing system using the detection and quantitation of polychlorinated biphenyls as a test case.

### An air to water bridge: air sampling and analysis using tetraglyme

*John R. Troost, Southern Petroleum Laboratories, Inc., 500 Ambassador Caffery Parkway, Scott, LA 70583, USA*

A water soluble organic liquid is shown to have the ability to scrub polar and non-polar, low and high boiling, halogenated and non-halogenated, as well as aromatic and aliphatic organic compounds from air and gas streams.

Air pulled through midget impingers containing chilled tetraglyme (an organic solvent used in USEPA Method 8240) is found to trap all Priority Pollutant, Hazardous Substance List and other volatile organic species with 100% efficiency. A portion of the tetraglyme is subsequently dispersed into water and analysed using gas chromatography/mass spectrometric Method 8240 (employing water standards) without modification.

Practical quantitation limits of 100 ppbv were demonstrated and the potential to achieve lower limits of detection is explored.

The method presents advantages over canisters, adsorption tubes, or tedlar bag air sampling techniques. Most notable are its general applicability, preservation of sample integrity (plating out of analytes is eliminated), freedom from water vapor interference, ready incorporation into standard water GC/MS methodology, simplicity and economy.

Environmental laboratories equipped with standard water analysis equipment can perform air monitoring analyses without special equipment or expertise.

### Development of a flow-injection spectrofluorometric analysis method for polycyclic aromatic compounds as a class in asphalt fume

*Charles E. Neumeister, Larry D. Olsen and Donald D. Dollberg, U.S. Department of Health and Human Services, National Institute for Occupational Safety and Health, Division of Physical Sciences and Engineering, 4676 Columbia Parkway, Cincinnati, OH, 45226, USA*

Recently, the US National Institute for Occupational Safety and Health established an Interagency Agreement with the Federal Highway Administration to investigate possible health effects associated with exposure to crumb-rubber modified asphalts. Previously, researchers have attempted chromatographically to analyse asphalt fume samples for select polycyclic aromatic hydrocarbons (PAHs). However, because asphalt fume contains numerous alkylated PAHs, the PAHs could not be resolved; consequently, almost no occupational exposure data was obtained. Also, many researchers believe the health hazards are associated with a variety of polycyclic aromatic compounds (PACs). For these reasons, a method was developed to evaluate PACs as a class.

Samples were prepared to allow separation of the PACs from the other compound classes in asphalt fume. Most sample preparation steps were automated to minimize the repetitive workload and provide greater precision. A flow-injection technique utilizing a high performance liquid chromatographic solvent delivery system, an autosampler, and two spectrofluorometric detectors was used to analyse PACs. The wavelength settings for the first detector provided greater sensitivity for the 2- and 3-ringed PACs, which are often irritants. While for the second detector, the wavelength settings provided greater sensitivity for the 4- and higher-ringed PACs, which are often mutagenic and possibly carcinogenic.

This flow-injection analysis method can be used to evaluate the PACs as a class; moreover, it can also be used to suggest possible health hazards. The method is sensitive, fast, and easy to use.

### Automated analysis of PAH in emissions with thermal desorption and GC/MS

*Gerhard Matz; Wolf M̈nchmeyer and Andreas Walte, Technical University of Hamburg-Harburg, Department of Measurement-Technology, Harburger Schloßstraße 20, D-27071 Hamburg, Germany*

The measurement of polycyclic aromatic hydrocarbon (PAH)-concentrations in stack emissions is time consuming and therefore expensive. To control emissions levels in short periods, or to get more information about the influence of combustion parameters quick measurements are needed.

The standard measurement of PAH is time consuming and can be divided into three steps: sampling, clean up and analysis. In this paper the application of thermal desorption, without further clean up steps, in combination with an automatically running analysis system based on gas chromatography/mass spectrometry (GC/MS) was described. With this combination analysis cycles of 5 minutes have been achieved on-site.

Aerosols are sampled from stack emissions with a null type probe and a dilution tunnel on a glass fibre filter tape. The sample is automatically transported to the desorption unit. This unit consists of two heated plates, which are moved by a pneumatic cylinder for desorption of the filter tape. During the desorption PAHs are injected into the column, which is plugged directly to this unit. The complex mixture of compounds is separated by the GC-column and identified by mass-spectrometry.

The thermal desorption unit is very simple and easy to integrate in an automatically running measurement system. However, the construction of the unit leads to different extraction efficiences for the PAHs with different boiling points. Correction is achieved by application of internal standard during the sampling process. By adding a known amount of standards, quantitative results can be achieved on-site.

The emission measurement system has been tested on different sites.

### Developement of an on-line TOC analyser for waste water monitoring

*Beverly I. Dulaney, Shimadzu Scientific Instruments, Inc., 7102, Riverwood Drive, Columbia, MD 21046, USA; Takeshi Iharada, Hiroshi Kitamura, Hidehito Nakagami, Yuko Sanji, and Minako Tanaka, Shimadzu Corporation, 1-Kuwabaracho, Nishinokyo, Nakagyo-ku, Kyoto 604, Japan*

The requirement for the on-line monitoring instrument is increasing in many fields, due to the global demands for the protection of the environment. For water quality monitoring, the Total Organic Carbon (TOC) is, in many aspects, considered to be very important index. The most important feature which is generally required for this monitoring instrument is durability against the dirty sample, which contains considerable amount of salt and/or suspended substances. Though the dilution of the sample prior to the measurement is the best way to reduce the trouble, it has required complicated configuration for the measuring system, such as the addition of an external diluter, etc.

An on-line TOC analyser was developed by the authors, taking into account above mentioned requirement. This instrument was specially designed to provide:

(1) Long-term stability with no service.
(2) Capability of analysing the sample containing salt and/or suspended substances.
(3) Multi-stream monitoring.
(4) Ability to measure TOC obtained by TC-IC, and NPOC with POC (optional attachment is required).
(5) Easy maintenance.

By using the multi-functional sample injector, sampling/injecting operation, and the sample pretreatment such as dilution, acidification, and sparging for IC removing is performed. The dilution function was successfully employed to reduce the interferences caused by the coexisting matrix and to make the lifetime of the catalyst, the combustion tube, etc. longer.

### Raman spectroscopy: from the laboratory to the process

*Bruce Chase, Corporate Center for Analytical Sciences, DuPont Experimental Station, PO Box 80328, Wilmington, DE 19880-0328, USA*

Raman spectroscopy has made tremendous strides in the last decade, with innovations that have turned an academic spectroscopy into a practical tool for industrial analyses. The use of both FT-Raman instrumentation and CCD detection, along with fibre optics has allowed the advantages of Raman monitoring to be utilized in a wide variety of environments. Measurements have been made on process stream, laboratory scale reactions, and in the fibre processing field. The orientation information available with polarized Raman scattering can be used very effectively in the latter. Both the FT-Raman and the CCD approach have specific advantages for process measurements, and a careful consideration of the specific problem is required to optimize the results. Several examples of process measurements using both techniques were given.

### Monitoring protein conformational changes by fast infra-red spectroscopy

*Olaf Weidlich, Holger Georg, Christoph Rödig, Christian Hackmann and Friedrich Siebert, Institut für Biophysik und Strahlenbiologie, Albert-Ludwigs-Universität, Albertstr. 23, D-79104 Freiburg, Germany*

The amide-I/II bands in the infra-red spectra of proteins are sensitive to their secondary structure. Therefore, monitoring these bands during biochemical processes allows the determination of structural changes. If the changes are very small, difference techniques have to be applied. It has been shown that infra-red difference spectroscopy is sensitive enough to detect molecular changes of single amino acid residues, such as protonation changes of carboxyl groups or environmental changes of aromatic residues. Since biochemical reactions are processes in time, time-resolved infra-red techniques have to be applied. In this contribution two methods were described. Both of them allow for a detector-limited time-resolution. With a photoconductive MCT detector it is approx. 500 ns.

With the so-called step-scan time-resolved FTIR spectroscopy systems can be investigated of which the reaction can be triggered many thousand times. The interferometer of the FTIR instrument moves in a step-wise manner and at each sampling point the static value and the time-resolved changes of the interferogram evoked by the reaction are measured. Usually 8 to 32 signals are averaged. This allows the detection of absorbance changes smaller than $10^{-4}$. Examples were discussed in which the method is applied to the light-driven ion pumps bacteriorhodopsin (BR) and halorhodopsin (HR) which both contain all-*trans*-retinal as chromophore. In the case of bacteriorhodopsin, time-resolved spectra of the native systems and of special mutants in which an amino acid in the retinal binding pocket has been replaced were compared. Possible intermediates in the photoreaction which differ in protein conformations but that cannot be distinguished by their absorption maxima in the UV-vis spectra of the chromophore were especially addressed. The elucidation of such intermediates is important for the understanding of the molecular mechanism of bacteriorho-dopsin. The time-resolved spectra of halorhodopsin were compared to the static low-temperature spectra obtained by stabilizing the respective intermediates of the photoreaction. This provides important information on the dynamics of the system. In addition, by adjusting the ionic conditions, the time-resolved spectrum of the HR-640 intermediate were presented. From this the structural changes occurring in this intermediate were derived.

Studies of the folding and unfolding pathway of proteins provide insights into the dynamics of the processes. Experiments were described in which unfolding and folding are evoked by a laser-induced temperature pluse. Since, in these transitions, larger structural changes were involved, an apparatus with monochromatic infra-red beam from a monochromator was used. The temperature pulse was induced by a Er: YAG-laser. Experiments on cytochrome-c was presented. They demonstrated that unfolding is a co-operative process involving a lag-phase of several tens of microseconds, and folding occurs on time-scale somewhat slower than 1 ms.

### The development of a field-portable instrument for the determination of lead azide

*Roger T. Echols, Division of Science & Mathematics, University of Minnesota Morris, 600 E. 4th St., Morris, MN 56267, USA; and Joseph H. Aldstad, Environmental Research Division, Argonne National Laboratory, 9700 S. Cass Avenue ER/203, Argonne, IL 60439, USA*

The contamination of the environment by explosives, such as lead azide, has led to an interest in the determination of expolsives in waters and soils and the monitoring of explosives on firing ranges and in ammunition storage and disposal areas. As with other environmental contaminants, there is a need for on-site quantitative methods of analysis for explosives and their degradation products. There is a requirement for lead azide, a primary explosive, to be determined by measurement of azide in order to avoid misleading results from other speciated forms of lead.

An amperometric method for the determination of lead azide using flow injection analysis techniques was presented. The goal of the research was the development of a field-portable instrument for the determination of lead azide in environmental samples. A flow injection system incorporating a BAS UniJet electrochemical cell was used in experiments to determine the concentration of sodium azide samples. The analyte is oxidized at a glassy carbon electrode at approximately $+1{\cdot}0$ V. Preliminary results indicate that the method has a lower limit of detection of 50 ppb and a linear calibration range of two orders of magnitude.

As a consequence of other anions that are common to environmental samples and that are oxidized at the analytical potential, the isolation of the analyte from the matrix will be an important consideration in the research. Ways to isolate azide from its matrix using ion-exchange columns and gas-permeable membranes were presented. The later method is attractive for separating azide from the matrix because of the relatively high vapour pressure of hydrogen azide.

### An expert system for the development of reversed-phase liquid chromatographic methods

*Robert W. Albrecht, L. R. Snyder and John W. Dolan, LC Resources Inc., 2930 Camino Diablo, Suite 110, Walnut Creek, CA 94596, USA*

In recent years, software to aid the development of liquid chromatographic (LC) methods has become widespread. Most of this software concentrates on using information from two or three pilot runs to help the user find the conditions that produce the best separation. Before such software can be applied, however, the user must make several decisions related to the method. The separation mode (isocratic or gradient), the column type, and mobile phase composition for the initial separation are usually chosen based either on personal experience of the user or on literature reports. The selection of initial instrument parameters is also left to the user. For the less-experienced user, or the experienced chromatographer using an unfamiliar method, this first stage of determining the starting point can be daunting. Further confusion can occur if the modelling software determines that a satisfactory separation cannot be obtained under the selected conditions. The authors discussed the development and application of an expert system that guides the user to the initial LC experiments and uses results from successful or failed experiments to move the user toward a satisfactory separation. The expert system starts out with a goal of a simple, binary separation, preferably in the isocratic mode. As additional dimensions of complexity are required to solve more troublesome problems, the expert system helps the user make logical choices so that gradient elution, ternary solvent systems, or other techniques can be applied to help solve the problem.

### Identification of paints using Curie-point pyrolysis/gas chromatography/mass spectrometry

*Ronald D. Snelling, OI Analytical, PO Box 9010, College Station, TX 77842-9010, USA*

Pyrolysis is one of the standard techniques for identifing polymeric materials. The material is decomposed at a high temperature, and the gas chromatograph trace is used to fingerprint the material. The most common technique in pyrolysis is the use of a resistively heated filament to decompose the material into smaller polymers and oligomers. The Curie-point technique uses a more reproducible technique where the temperature of the filament is a function of the composition of the filament's alloy.

The utility of the Curie-point pyrolysis technique was demonstrated by the analysis of a variety of paints. The fingerprint of each type of paint was different, and the various pigments in different colours of paint also may be used for further identification of paints. Curie-point pyrolyzer/gas chromatograph/mass spectrometry chromatograms for a number of paint samples were shown, illustrating how the technique may be used to identify unknown paints.

### Innovative automation software for FT-IR spectroscopy applied to industrial problem-solving

*Michael C. Garry and John C. Bowman, Nicolet Instrument Corporation, 5225 Verona Road, Madison, WI 53711, USA*

Implementing routine Fourier transform infra-red (FT-IR) quantitative methods in production environments is a complex task, even for a highly skilled chemist. Details of quantitative method development sample handling, operator interface, result output formatting and liking to information management systems all provide challenges in the quest for a robust, reliable, and useful analytical procedure. Most of the above details are very similar between a wide variety of analytical methods, so a software template can be created to provide a consistent operator interface and reporting structure. However, this template must have inherent flexibility to allow a development chemist access to customize the features for their particular needs without forcing complex programming to be done.

This presentation focused on automation software for an FT-IR spectrometer that has been used to solve routine analysis problems within industrial settings. The software uses a Windows based operator interface that is easy to use while at the same time being very flexible. Included in the procedures are safety and analysis quality checks along with intelligent quantification methods featuring programmed warning messages. Examples of industrial problem-solving were presented, including used lubricant analysis and edible oil process monitoring.

### A new fully automated solution for the analysis, confirmation, and reporting of results for the drugs of abuse in biological fluids and tissue

*Wayne S. Miles, Lenore G. Randall, Hewlett-Packard Company, 2850 Centerville Road, Wilmington, DE 19808; and Amal Dasgupta, Delaware Medical Examiner's Office, 200 South Adams Street, Wilmington, DE 19801-5104, USA*

The need to analyse different biological fluids for the drugs of abuse is increasing. This presents some added problems to the analyst whose schedule is already overly taxed. Thus the ability to automate some or all of the tasks surrounding the analysis is a well received instrument feature. Another desirable feature of the automated approach is the ability to automate already validated manual methods. This offers the advantage of considerable decrease in methods development time which provide short start-up times.

In this paper the authors presented results of the application of a fully automated approach that couples sample preparation and analysis with a customizable report generating software package. This approach differs from more traditional approaches, in that it offers a total solution for those laboratories performing extraction, conformation and analysis of the drugs of abuse. Authentic specimens are automatically analysed in a batch mode with sample-tracking and reporting capabilities of individual and batch summary reports for compliance. A just-in-time approach to sample preparation readies subsequent samples for analysis in a time comparable with the analytical instrument cycle time.

This approach was validated using authentic specimens and the results were compared to those obtained on split samples using traditional manual methods. Automated recoveries for the drugs studied in urine, blood, serum, and post-mortem tissue were comparable to manual results. Precision results for the automated approach were found to be considerably better with overall % CVs in the range of 0·68 to 5·98, and 1·57 to 8·46, for low and high concentrations of drug, respectively. Other than better quality results, some additional benefits of this approach are: (1) a decrease in user hands'-on time; (2) decrease in analysis cost/sample; (3) a reduction in exposure to hazardous chemicals and samples; and (4) fully unattended operation.

### Development of an automated laboratory workstation to prepare and inject solid samples for HPLC analysis

*Patricia M. Ahrweiler and James R. Harness, Bohdan Automation, Inc., 1500 McCormick Boulevard, Mundelein, IL 60060, USA*

An automated laboratory workstation has been developed that performs the sample preparation steps and the sample injection for performance of HPLC analysis on solid samples. The workstation adds solvent to the samples, performs an extraction by vortex mixing, dilutes the sample to the selected concentration, filters a portion of the diluted extract through disk filters, and injects the sample into an HPLC unit. Standards stored on the workstation can be injected at selected intervals to bracket sample injections. All sample extracts, filtrates and dilutants are held in individual containers to eliminate the concerns of carry-over and cross-contamination associated with repeatedly transporting solutions through common vessels, lines and valves. The workstation consists of a covered rack for standards/controls; racks for sample tubes, filtrate tubes, dilution tubes, and filters; two vortex mixers; grippers; filter remover; a leur-tipped cannula; and a cannula wash station.

The workstation capacity is 30 samples per run with a throughput of approximately 12 minutes to process a sample through injection. Liquid handling is accomplished using syringe drives and Teflon, glass and Hastelloy materials. Pipetting accuracy/precision for the dissolution and dilution processes are 99·7/1·3% and 99·1/1·0%, respectively. Therefore, a workstation has been developed that can handle solids processing for HPLC analysis with a high degree of accuracy and precision without concerns about carryover and cross-contamination.

### Ruggedness testing: comparison of automated instrumentation for content uniformity testing

*Patricia M. Young, Raymond P. McGuirk, Waters Corporation, 34 Maple St., Milford, MA 01757, USA; and Susan Kelley, Source For Automation, 327 Fiske Street, Holliston, MA 01746, USA*

US regulations require content uniformity and composite assay testing for all solid dosage forms manufactured or imported into the US. Ordinarily, these tests are done manually with labour spent weighing and diluting samples, dispersing the solid dosage form matrix, and maintaining accurate records. The requirement for high throughput and comprehensive management of the sample preparation process, tablet weights, dilutions, content analysis and data reporting to complete the audit trail for GMP/GLP compliance has made automation essential. Typically, analytical methods are developed at one site and transferred world-wide to quality control or quality assurance laboratories at manufacturing sites. User expertise and laboratory equipment at development sites may be different to those at manufacturing sites. With rugged automation of the content uniformity process from sample preparation to analytical HPLC and results management, we can ensure that methods will be faithfully transferred.

All segments of tablet processing, from sample preparation to component quantification, can be accomplished with the Waters Tablet Processing System. While spectrophotometric analysis may be effective for dosage forms having a single component, increasingly, purity assessment and drug stability assays are required. These tests, combined with the need to quantify all ingredients in a multi-component drug, have increased the necessity for HPLC separations. WTPS provides a validated platform for automated continent uniformity testing, component quantification and results management.

### Flow injection analysis of mixtures of the triazine herbicides simazine, atrazine and propazine using filter-supported Bilayer Lipid Membranes (BLMs)

*D. P. Nikoleilis, V. G. Andreou and G. C. Siontorou, Laboratory of Analytical Chemistry, Department of Chemistry, University of Athens, Panepistimiopolis-Kouponia, 15771-Athens, Greece*

Biosensors for the detection of trazine herbicides are mainly based on photosynthetic systems or antibodies, however, these sensors cannot operate as continuous control systems and lack selectivity to distinguish between trazines. The authors' present research is focused on the use of bilayer lipid membranes (BLMs) for the selective monitoring of environmental pollutants.

Conventional planar 'free-suspended' BLMs were found to electrochemically respond to triazine herbicides providing a transient current signal as a single event which was linearly related to triazine concentration, and a technique for the continuous flow monitoring of simazine, atrazine and propazine using filter-supported BLMs was developed. The mechanism of signal generation was studied electrochemically, and through differential scanning calorimetry experiments of lipid vesicles. The mechanism of signal generation has shown to occur in two discrete steps: a fast adsorption of the trazine in BLMs, followed by a consequent rate-determining aggregation phenomenon of the herbicide at the surface of the membrane. The reversibility of the response allowed a large number of injections without degradation of signal magnitude. Furthermore, the time delay of signal appearance increased in line with the order of simazine, atrazine and propazine, therefore allowing a selective determination of each triazine in mixtures.

The present technique holds prospects of flow injection analysis of these triazine herbicides in mixtures to replace chromatographic procedures having advantages of continuous analysis in the field at low cost in less than two minutes.

### On-line denaturation/capillary microreactors/capillary zone electrophoresis for generation, separation and detection of protein digests

*Larry Licklider and Werner G. Kuhr, Department of Chemistry, University of California at Riverside, Riverside, CA 92521, USA*

Peptide mapping characterization of proteins generally consists of many intermediate procedures to denature a protein for greater accessibility peptide bonds, followed by removal of denaturants and the application of a specific enzyme catalyst to generate peptide fragments which are utilized for structural characterization. Fused-silica enzyme capillary microreactors (20 nl or smaller volumes per cm length) containing a variety of proteolytic enzymes immobilized at the inner capillary surface are especially well suited for on-line peptide mapping by CZE of low picomole quantities of proteins. The feasibility of transferring nanoliter or smaller aliquots via a fluid capillary junction was examined by spectroscopic imaging of a highly absorbing dye. Optimization of the microreactor digestion of globular proteins has recently been demonstrated using mechanical and piezoelectric transducers. The frequency of the piezoelectric transduction has been varied over a wide range to increase the rate of conversion of globular proteins to their tryptic fragments. The feasibility of denaturing low picomole quantities has been investigated in an on-line ultrafiltration cell (30 nl/mm) constructed of several mm of a hollow fibre dialyser (180 mm i.d.) joined with short segments of fused-silica capillary (50 mm i.d.). The ultrafiltration cell was demonstrated to remove low molecular weight solutes from the cell within several minutes.

### Optimizing automated micro-derivatization for HPLC analysis of amines

*Michiel H. Godschalk, J. Bert Ooms, Spark Holland B. V., PO Box 388, 7800 AJ, Emmen, The Netherlands; and Günes Barka, SunChrom GmbH, Max-Planck-Str. 22, D-61381, Friedrichsdorf, Germany*

Automated pre-column derivatization of amino acids with Orthophtal-dialdehyde (OPA) or Fluorenyloxycarbonyl-chloride (FMOC) using an HPLC autosampler with reagent addition capability has become a common procedure in the bioanalytical laboratory. However, the continuing demand for miniaturization of analytical methodologies to allow analysis of the available amount of sample is now pushing conventional instrumentation to its limits or beyond. As a typical example, microdialysis fractions collected for HPLC analysis are in the order of 10 to 50 μl with a definite tendency toward volumes in the range of 1 to 5 μl.

In this paper the authors presented procedures and hardware for automated pre-column derivatization of amino acids with OPA and FMOC in sample volumes ranging from 1 to 50 μl using a Triathlon autosampler. Common reagents or ready to use reagents kits as described in the literature were used. Liquid handling and reagent mixing procedures were optimized in relation to sample volume and vial geometry. With the optimized procedure, reproducible derivatization and injection of an amino acid standard is possible with RSD values in the 1–3% range. Examples of applications in bioanalysis were also shown and discussed.

### High performance liquid chromatography-inductively coupled plasma-mass spectrometry (HPLC-ICP-MS) for measurement of arsenic species in environmental samples

*Dean A. Bass, Judith S. Yaeger, James T. Kiely, Jeffrey S. Crain, Analytical Chemistry Laboratory, Chemical Technology Division, Argonne National Laboratory, 9700 S. Cass Avenue, Argonne, IL 60439, USA; Mark J. Gowdy, Linda M. Shem, Hugh J. O'Neill, Energy Systems Division, Argonne National Laboratory, 9700 S. Cass Avenue, Argonne, IL 60439, USA; Greg B. Mohrman and Mark Besmer, Rocky Mountain Arsenal, Building 130, Commerce City, CO 80022-2180, USA*

A method has been developed by Argonne National Laboratory to identify and quantify As(III), As(V), and organoarsenic compounds in soil and water samples. Soil samples were spiked with arsenic oxide, sodium arsenate, dimethyl-arsinic acid (DMAA), and chlorovinyl arsenious acid. The arsenic species in these soils were extracted and then identified and quantified with the HPLC-ICP-MS system. The arsenic species were extracted from the soils by using the HPLC mobile phase and sonication. Extraction efficiencies for various extraction fluids and the stability of the arsenic species during the extraction process were presented. The arsenic species were separated by reversed-phase, ion-pairing HPLC using a microbore column and were introduced into an ICP-MS system by a direct injection nebulizer. Detection limits were 0·1 pg As (as injected on the column) for each arsenic species. Chromatography and ICP-MS conditions were described, as were quality control measures, for example the use of check standards, control samples, surrogates, internal standards, and matrix spikes. Precision and accuracy information was presented from the analysis of aqueous standards and soil extracts.

Soil samples were analysed by HPLC-ICP-MS in support of the analytical needs of thermal desorption treatability studies being conducted at the Rocky Mountain Arsenal for chemical warfare agents and pesticides. Arsenic species in contaminated soils were measured before and after thermal treatment. These data demonstrate the utility of this arsenic speciation method for the sensitive determination of arsenic species present in soil or water.

### Flow injection techniques for interference removal in plasma source mass spectrometry

*Paul Becotte-Haigh, Julian. F. Tyson, Department of Chemistry, University of Massachusetts, Box 34510, Amherst, MA 01003-4510, USA; Eric Denoyer and Susan Mcintosh, Perkin-Elmer Corp, 761 Main Avenue, Norwalk, CT 06859, USA*

There are a number of well-known interferences in plasma source mass spectrometry which arise form the presence of matrix components. These are due to spectral overlap and to transmission changes in the ion lens system due to the effect of other ions. There are several possible approaches to overcoming such effects. In principle, the most effective of these is the separation of analyte and matrix prior to introduction to the spectrometer. Flow injection procedure provide a practical means of implementing some separations that would be tedious to perform in a routine manual situation such as precipitation and solid phase extraction.

To illustrate the scope of flow injection in this regard, several methods have been developed for the measurement of arsenic in gold at concentrations down to 1 ppm in the solid. Gold has been separated by batch precipitation and by on-line extraction with an anion ion-exchange resin. There was good agreement between results obtained by these methods in conjunction with hydride generation and with results obtained by the Royal Canadian Mint by graphite furnace atomic absorption spectrometry for a range of sample concentrations between 1 and 50 mg/kg. Preliminary results for the solid phase extraction removal of uranium matrix with subsequent multielement determination of light elements were also presented.

### Single standard calibration and on-line over range sample dilution in hydride generation atomic absorption spectrometry

*Doug Shrader, Varian Optical Spectroscopy Instruments, 201 Hansen Court, Suite 108, Wood Dale, IL 60191, USA; Jonathan Moffett and Eric Vanclay, Varian Australia Pty Ltd, 679 Springvale Road, Mulgrave, 3170, Victoria, Australia*

Calibrating a flame Atomic Absorption (AA) spectrometer usually requires preparation of a range of standards which are aspirated in turn to measure the absorbance. Different approaches to simplifying calibration have been discussed. To date, the only commercially available system that dramatically simplifies calibration for flame AA is the Varian Sample Introduction Pump System (SIPS). The patented SIPS has eliminated preparation of multiple calibration standards and pre-dilution of samples with fast, accurate on-line dilution. This has also dramatically improved the productivity of flame AA determinations.

Using SIPS, the flow rate of sample delivered to the nebulizer is directly proportional to the speed of the pump. The dilution ratio applied to the solution being introduced is precisely controlled using computer controlled stepper motor driven peristaltic pumps. Peristaltic pumps also form the basis of most commercially available hydride or vapour generation systems, which use peristaltic pumps to pump the sample, an acid and a reductant. These solutions are then combined using specialized plumbing initiating the reaction that forms the volatile metal hydride. The hydride vapour is separated from the waste solution and passed to a heated absorption cell positioned in the optical path for measurement.

This paper discussed the technical aspects of adapting the SIPS to form the basis of a hydride generation system. Practical operation of the system, including calibration from a single standard and on-line over range sample dilution was demonstrated.

### Automated sample preparation and thin layer chromatographic (TLC) analysis of acid, base and neutral drugs and their metabolities in multiple urine samples

*Roger Quinn Roberts, ANSYS, Inc., 2 Goodyear, Irvine, CA 92718, USA*

Performance of a new device (TOXI·PREP, ANSYS, Inc., Irvine, CA) which incorporates a vacuum manifold system for sample extraction and a heated (115 °C) application zone for TLC was presented. The device extracts up to seven samples using solid phase extraction (SPE) columns (SPEC·, ANSYS, Inc.) and applies the eluates of those extracts directly to a special TLC plate (TOXI·GRAM, ANSY, Inc.) for the differential analysis of acid, base and neutral drugs and their metabolities. One hundred 10·0 ml urine samples were each treated with 1·0 ml of 1·0 M acetic acid. Seven SPE columns were inserted into the device's vacuum manifold and conditioned with 200 µl of isopropanol and 1·0 ml of 0·1 M acetic acid. Samples were applied to the SPE columns and a TLC plate was placed on the heated application zone. TOXI·PREP controlled sample extraction and subsequent extract deposition onto the TLC plate. The analyst followed a written extraction procedure and activated the appropriate TOXI·PREP control at each step. Upon completion of the extraction, the TOXI·PREP device positioned the SPE columns over the TLC plate causing their Luer tips to seal onto the TLC plate. Sequential applications of acidified ethyl acetate and ammoniated ethyl acetate (1·0 ml) were applied to the SPE columns and the eluates were automatically applied to two separate TLC plates by evaporation of the eluates at the TLC plate/SPE column Luer tip interface. As each elution was completed, the TLC plate was removed for development and drug detection. One hundred samples were analysed for 40 different acid, base and neutral drugs and their metabolities in approximately 5 hours. Limit of detection ranged from 50–2000 ng/ml.

### Applications of automated flow injection analysis to quality control in fertilizer, beverage, tobacco, and chlor-alkali industries

*Kevin Switala, Lachat Instruments, 6645 West Mill Road, Milwaukee, WI 53218-1239, USA*

Flow injection analysis (FIA) is an automated analytical technique which is a valuable addition to quality control laboratories. FIA frees the chemist from routine manual handling of samples and reagents and it reduces the possibility of error due to different operators.

FIA is used in the analysis of liquid fertilizers and the water extracts of dry fertilizers for ammonia, nitrate, urea, and potassium. Wine and soda production are areas in the beverage industry where FIA can analyse multiple analytes and free chemists for other tasks. Total sulphur dioxide, free sulphur dioxide, total acidity, and volatile acidity, can be determined in wine. Phosphate is determined in soda. In the tobacco industry, FIA detects ammonia, nitrate, urea, and potassium sorbate in tobacco extracts. The chlor-alkali industry produces chlorine and sodium hydroxide from brine solutions with electrolytic cells. The chlor-alkali industry is interested in the determination of sodium chloride, sodium hydroxide, sodium chlorate, and sodium hypochlorite in order to increase current efficiency in the electrolytic cell. Four methods have been developed for the determination of the previously mentioned analytes.

The chemistries used in the industrial quality control applications were discussed, along with a description of gas diffusion and dialysis FIA techniques which were incorporated in order to solve specific problems. The chemistry manifolds, calibration, and precision data were also presented.

### Considerations in moving FT-IR spectroscopy from the laboratory to the plant environment

*Jay Powell, Roger Gervais, David Dellea and Andrew Grady, Bio-Rad Laboratories, Digilab Division, 237 Putnam Avenue, Cambridge, MA 02139, USA*

FT-IR spectroscopy has grown from a general purpose, advanced research tool to a routine analytical instrument. The desire to reduce the turn-around time from when a sample is acquired to a final decision on an action to take (based on the analytical results) necessitates moving FT-IR spectroscopy from the laboratory environment to less-than-ideal locations such as the plant production floor or the shipping and receiving dock. While hardening of the spectrometer optics and electronics (rugged interferometers, power line filtering and UPS, environmentally appropriate NEMA enclosures, etc.) is usually the first step taken in this transfer, there are other factors which have a greater influence in determining the success of this move. Ultimate success is determined by not only how rugged the hardware is to the environment, but also by how well the complete transfer of the hardware and the application is made into this environment.

First, consideration must be placed on the infra-red sampling technique. As the target operator typically is not an infra-red spectroscopist, or perhaps even a chemist, the sampling technique must be simple and easy enough to allow an untrained operator to properly present the sample to the spectrometer with minimal preparation, and insensitive to the unavoidable variations from one operator to the next. ATR has typically been used in this application, as a consistent infra-red sampling pathlength is assured by a fixed crystal size, fixed composition, and fixed IR transfer optics. For solid samples hardware to apply a consistent pressure between the sample and the crystal has become relatively common. However, not all samples lend themselves to ATR sampling. Other techniques, ranging from classical transmittance sampling through bulk specular reflectance, have been employed depending on the characteristics of the samples to be analyzed.

Second, general purpose FT-IR spectrometers are equipped with a wide variety of software parameters and options, such that the spectrometer can be configured for a variety of experiments. However, analysis parameters needed at the point of sampling in a production environment are typically more restricted. Not only do the general purpose spectrometer parameters need to be fixed and hidden from inadvertent modification, but the analysis routines must be simple enough for an operator with minimal training to understand and it must also allow routine mistakes. While these parameters should be hidden from the routine operator, there is most often several additional levels between the routine operator running the analysis and the analytical chemist responsible for the development and implementation. These intermediate levels may have the knowledge and requirement to access these parameters, and adapt them as the immediate situation dictates. A successful transfer of IR technology from the laboratory to production environment should take the needs, training, and background of these personnel into account to ensure their responsibilities can be performed with the system as well as the routine operator. Typically, this has been achieved through custom programming for each implementation. Custom programming adds significant expense to the implementation, and what is needed is a hierarchical template which aids in the implementation for these different levels.

Finally, consideration must be paid to the destination and use of the information. This information can range from a simple pass/fail message presented to an operator, who takes appropriate action, to a detailed analysis report sent to a remote server for integration with other reports from other sample tests and monitors. Once again, there may be additional levels of supervision or management which this information must pass, and be judged in, before it reaches its final destination. As before, this has typically been done through custom programming, while what is needed is set of selectable, flexible reporting output formats which will be useful to both humans and other computer systems.

The authors reviewed several FT-IR applications that have been successfully moved from the analytical method laboratory to a production environment. The factors discussed above were used to explain considerations in successful method transfer and implementation. The common factors across these applications were then presented as a general guide towards designing and implementing production level infra-red analysis.

### Fuel spill identification by high speed gas chromatography/pattern recognition techniques

*Barry K. Lavine, Jason Ritter, Department of Chemistry, Clarkson University, Box 5810 Potsdam, NY 13699-5810, USA; Abdullah Faruque and Howard Mayfield, AL/EQ, 139 Barnes Drive, Suite 2, Tyndall AFB, FL 32403-5323, USA*

The possible contamination of groundwater by fuels stored in leaking underground tanks and pipelines has prompted the development of new methods for identification of fuel materials recovered from subsurface environments. Recently, the authors have investigated the potential of head space solid phase microextraction as a method to relate an underground fuel spill to its source. Solid phase microextracts of fuels which had undergone weathering in a subsurface environment were correctly identified as to type using discriminants developed from the gas chromatograms of unweathered jet fuels. This approach to fuel spill identification has been taken because the physical and chemical interactions of jet fuel components with the subsurface environment are not well understood.

### New instrumentation for on-line bioprocess monitoring incorporating amperometric biosensors

*A. P. F. Turner, J. D. Newman, S. F. White, I. E. Tothill, Cranfield Biotechnology Centre, Cranfield University, Cranfield, Bedfordshire MK43 OAL, UK; and J.-C. Robert, Gilson Medical Electronics, 72 rue Gambetta, B.P. 45, 95400 Villiers-le-bel, France*

Bioprocess control would benefit from the rapid on-line analysis of as many significant parameters as possible. Conventionally, flow meters, pH probes, oxygen electrodes and temperature sensors are used in fermentation control. Despite considerable interest in expanding this list, commercial products are scarce. Two significant reasons for this are the difficulty in operating in such variable and complex matrices and the problem of manufacturing suitable chemical sensors. The authors reported on a fully-integrated instrument, consisting of a sampling unit, a microprocessor-controlled flow-injection system and a mass-producible enzyme electrode which is initially being marketed as a glucose analyser.

The biosensor is produced by screen-printing a multi-layer, three electrode planar array consisting of a carbon counter electrode, a silver/silver chloride reference electrode and a catalytic carbon working electrode. The enzyme, glucose oxidase, is mixed with the working electrode material, which is based on powdered rhodinised carbon, in a water soluble polymer binder. On top of this is printed a membrane which prevents the working electrode, including the enzyme, from being washed away, yet enables the whole structure to be hydrated. In addition, the membrane acts as a diffusion barrier, extending the linear range of the device and excluding large interferent molecules. The rhodinised carbon powder selectively reduces the potential required for the oxidation of hydrogen peroxide, offering a considerable advantage over materials such as platinised carbon. At the operating potential of +350 mV, high current densities are obtained with little interference. A further advantage is that this voltage lies in the middle of a response plateau, which means that small fluctuations in the reference voltage have little effect on the current. The design is such that it is amenable to adaptation to alternative analytes. These could include lactate, ethanol, glutamate or other metabolities which are relevant to bioprocess monitoring.

The flow-injection unit allows up to 12 measurements to be made per hour and contains a temperature measurement and compensation system. It also facilitates automatic sample dilution with a buffer solution, which contains chloride ion for the reference electrode, such that a wide range of concentrations can be monitored. In the case of glucose, this range is 0·5–1000 mM. In addition, there is a built-in, periodical calibration step which compensates for the gradual loss of enzyme activity over time. Hence, the sensor can be operated for at least seven days before the card needs to be replaced. This feature differentiates this biosensor from previous screen-printed devices, which have tended to be restricted to 'single-shot' use.

### At-line evaluation of pharmaceutical pellet characteristics during rotogranulation by near-infrared spectroscopy

*David J. Wargo, James K. Drennen, Division of Pharmaceutics, Graduate School of Pharmaceutical Sciences, Duquesne University, Pittsburgh, PA 15282, USA; and Francis X. Muller, Mylan Pharmaceuticals, Inc., Morgantown, WV 26505, USA*

Application of drug onto non-pareil seeds by rotogranulation is one of several important steps in the production of multiparticulate controlled release pharmaceutical dosage forms. Near-infra-red (near-IR) spectroscopy was used to monitor the potency of drug-layered spheres at-line during laboratory and production scale rotogranulation processes.

A water soluble drug, water soluble polymer, and anti-tacking agent were layered onto non-pareil seeds using rotogranulation fluid-bed equipment. Unit-dose samples were collected at numerous time points during processing and scanned at-line using a grating-type near-IR spectrometer. Changes in near-IR spectra were evident as the amount of applied drug was increased.

After obtaining near-IR spectra for each sample, potency of the drug-layered spheres was determined using HPLC. Principal component regression allowed development of quantitative models for near-IR determination of potency. Using the established calibrations, it was possible to accurately predict pellet potency at-line during laboratory and production scale rotogranulation operations and achieve reproducible product characteristics at respective process endpoints.

### Flow injection electrochemical hydride generator for atomic spectrometry

*M. S. Jimenez, F. Laborda, J. M. Mir and J. R. Castillo, Analytical Spectroscopy and Sensors Group (G.E.A.S.), Department of Analytical Chemistry, Faculty of Sciences, University of Zaragoza, 50009 Zaragoza, Spain*

Hydride generation combined with atomic spectrometry is an established technique in the determination of As, Bi, Ge, In, Pb, Sb, Se, Sn, Te and Tl. The generation of these volatile hydrides can be performed in three different ways: chemical, thermochemical and electrochemical. Chemical generation is mainly based on the reaction of the analyte with sodium tetrahydroborate in acid medium. Hydrides can be generated thermochemically by mixing the analyte with hydrogen in a thermospray at high temperature. Electrochemical generation is based on the formation of hydrogen and the hydride in the cathode of an electrolytic cell, whereas oxygen is being produced in the anode, acid solutions are used as anolyte and catholyte. This option offers several advantages; in addition to acid no other reagent is required, generation is performed at ambient temperature, and interference from transition metals can be eliminated.

Recently, two flow injection generators based on thin layer electrolytic flow cells using platinum electrodes have been described. In this work, tubular electrolytic flow cells are designed as an alternative to thin layer ones and the use of graphite based electrodes is tested. Performance of both types of electrolytic flow cells as electrochemical hydride generators were compared in this paper.

### The monitoring of hydrazine and monomethyl hydrazine degradation by ion chromatography

*Terry L. Thiem, Polymer Research Division, Bayer, New Martinsville, WV 26155, USA; E. Holwitt and J. Kiel, Armstrong Laboratory, San Antonio, TX 78235-5324, USA*

Hydrazine, monomethyl hydrazine, and unsymmetrical dimethyl hydrazine are used extensively by the US Air Force and NASA as a bi- or mono-propellent in rockets, satellites, and the Space Shuttle, as well as in alternate power units in F-16 jet fighter aircraft. Unfortunately, hydrazines can enter the environment through spills during fuelling of the rockets or plane crashes and they become an environmental hazard. A study was undertaken at the Air Force Academy's Chemistry Department to evaluate possible methods of degrading the hydrazine once it entered the environment.

Derivatization for methods using HPLC and UV/VIS were unreliable due to interferences with by many of the different transition metal catalysts. Ion chromatography was tested and found to have little, if any, interference from the catalysts and therefore was the method of choice for the study. Degradation information from the ion chromatography data was presented, as well as instrumental parameters and detection limits for both hydrazine and mono-methyl hydrazine.

### The determination of arsenic +3 and arsenic +5 in contaminated ground water utilizing ion chromatography with conductivity and pulsed amperometric detection

*Janet E. Heitert and Pradeep Chheda, Environmental Research Institute, University of Connecticut, Rr. 44 Longley Building, Storrs, CT 06269-3210, USA*

In this paper, the authors described the analytical protocol for an ion chromatography method for the determination of As +3 (arsenite) and As +5 (arsenate) in contaminated ground water from a Superfund site. The characterization of ground water is necessary prior to determining which treatability studies will be undertaken and later employed in physical/chemical treatment technologies to effect successful remediation programmes.

A Dionex 4000I ion chromatograph, equipped with a conductivity detector for the determination of arsenate and a pulsed amperometric detector with platinum electrode for the determination of arsenite was utilized in this procedure. Anion exchange columns Dionex AS12A and AG12A) with micro-membrane suppression and a carbonate bicarbonate eluent were used to separate arsenic +5. The ion exclusion column Dionex ICE AS6 and a dilute sulphuric acid/acetonitrile eluent was used to separate arsenic +3.

Since the ground water samples contained various organic arsenic species and a heavy concentration of inorganic ions, the matrix was complex and the procedure required many adjustments to eliminate the resulting interferences, such as oxalate coeluting with arsenate. Varying eluent concentrations and flow rates resolved the problems encountered. Sample handling and preparation and operating conditions and settings for the ion chromatograph and delactors were discussed, along with a comparison of ions chromatography data and inductively coupled plasma (ICP) results for total arsenic.

### Strategies for process monitoring by NIR spectroscopy

*Douglas L. Martin, Steve Brezina, Thomas Kapral and Donald E. Muller, Bran + Luebbe, Inc., 1025 Busch Parkway, Buffalo Grove, IL 60089-4516, USA*

There are two common views of process control. One is the analysis of the finished product to check that it meets specifications. Another is a black box instrument which gives a relatively continuous quantitative analysis of one or more constituents at some process point. Near infra-red (NIR) spectroscopy has served well in these two functions.

There are many other ways to use rapid non-destructive NIR analysis for process monitoring and control. Anywhere that raw materials are added to the process, NIR can be used to identify and quality them as the proper ingredient. Mixing homogeneity can be tested both in batch mixers or in flowing systems. Reactions can be followed to ensure completion before the next process step. Drying and/or solvent removal can be followed with NIR.

Instrument and software developments have added new NIR techniques for production monitoring and control. Different NIR instrument technologies and sample presentation techniques contribute to the range of useful control strategies. Applications were presented to illustrate a wide variety of control strategies made more effective with NIR.

### On-line analysis of pharmaceutical mixtures using Raman and PLS

*R. M. Belchamber. T. Lilley, M. P. Collins, Process Analysis & Automation Ltd, Falcon House. Fernhill Road. Farnborough, Hampshire, GU14 9RX, UK; and Ruth Geiger, Dilor GmbH. Wiesenstr., 4, D-64625 Bensheim. Germany*

This paper described the development of an on-line, real-time multi-component analysis that uses a combination of Raman spectroscopy and Partial Least Squares (PLS) regression for the quantitative determination of caffeine, ascorbic acid and paracetemol in a mixture. Raman spectroscopy has great potential as a non-invasive process monitoring technique but has been restricted by high cost of on-line implementation and the non 'process hardened' nature of the apparatus. However, recent developments in the hardware (such as Diloris INDURAM spectrometer) are now enabling Raman systems to be used in the process environment in direct competition with the relatively well established near infra-red spectroscopy. Raman has more discriminating power because it operates in the fundamental vibration region of the spectrum, unlike the near infra-red region, where overtones and combination bands exist.

PLS is a powerful mathematical technique that enables the user to infer one set of properties of a system from another—in this case the chemical composition of a pharmaceutical mixture in aqueous solutions from Raman spectra. For on-line process analysis, this approach allows users to effectively by-pass rigorous spectroscopic analysis and measure the component concentrations directly. The benefits to be gained from PLS will not be fully realized until it can be seamlessly integrated into control and monitoring systems making the techniques available to process engineers on-line. The authors have developed a LabVIEW tool kit. CharmPLS, with this purpose in mind. LabVIEW, from National Instruments, is rapidly emerging as a first choice for process control and monitoring system development. By using the tool kit, chemists have the ability to develop powerful real-time instruments on-line that measure directly the concentration of key process components.

Twenty-two mixtures of caffeine, ascorbic acid and paracetemol across the range 0·01 to 0·1 mol $dm^{-3}$ were prepared and their Raman spectra were recorded (200–1500 $cm^{-1}$). A spectral reduction of 1 to 3 (i.e. 489 data points) was used to reduce computational overhead and improve signal to noise. These were analysed with the PLS algorithm to provide a PLS multi-component calibration using the PLS-2 algorithm. Models were produced with a correlation of better than 0·97. PRESS (Prediction Residual Error Sum of Squares), a cross-validation technique, was used to optimize the PLS model. The PLS models were generated off-line using this test set. As this stage is off-line the predicted data set can be obtained using any number of complex and time consuming procedures enabling the user to eliminate the works laboratory from process monitoring. Once modelled these are used for on-line prediction. If process specification changes, the additional calibration data can simply be added to the model.

### On-line quantification of pharmaceutical fermentation components by near-infra-red spectrometry

*Andrew Lange, Tom Hoerner, Rob Kasprow, Eric Richmond and Tim Schimmel, Merck & Co., Inc., Route 340 South, Elkton, VA 22827, USA*

Near-infra-red (NIR) spectrometry has been shown to be a quick, non-destructive way to monitor chemical processes. The work described in this presentation applied this technology to the area of bioprocess monitoring. Data were presented from two different microbial fementation systems which produce bulk pharmaceuticals. The main advantages of this on-line method are: improved process control leading to decreased fermenter turn-around time, reduced waste and product loss due to sampling, and reduced sterility concerns due to the non-intrusive nature of the measurement.

NIR probes were inserted into both pilot and production-scale fermentation vessels. These probes consist of a fibre optics bundle encased in a stainless steel sheath terminating with a quartz window. Diffuse-reflectance NIR spectra were taken, and several components were quantified in real time. Data treatment and temperature effects were discussed.

### On-line process monitoring of gas streams by FT-IR spectrometry

*Deru Qin and Gardy Cadet, AT&T Bell Laboratories, Room 1A-372, 600 Mountain Ave, Murray Hill, NJ 07974, USA*

The combination of accuracy and speed of measurement coupled with statistical analysis makes FT-IR spectrometry an invaluable tool for a wide range of applications in process monitoring and control.

Quantitative determination of the composition of gas phase effluent streams following high temperature chemical processing is of paramount importance for on-line monitoring of reaction by-products, process control, and process end-point detection. In this work, a low-resolution FT-IR spectrometer was developed for simultaneously determining the chemical components and species composition evolved from the high temperature processing of organic-containing solid materials. Low resolution spectrometry provides high speed, low cost, and good signal-to-noise ratio of spectral data for quantitative process analysis. The concentration of each component in the sample was determined by a partial least squares (PLS) regression method. IR spectra of pure component mixtures obtained from 4000–700 $cm^{-1}$ at a resolution of 16 $cm^{-1}$ were used as calibration standards for the PLS model. Quantitative data on the concentration of individual components was obtained by fitting the sample spectra to the calibration model. The accuracy for water determination obtained by this method is found to be greater than 99%. The root mean squared deviation for the component showing a significant overlapped spectral feature is less than 1%. FT-IR spectrometry combined with statistical analysis has been shown to provide a rapid, accurate, and reliable methodology for studying kinetics and reaction by-products of high temperature chemical processes.

### On-line analyser for total fluorides in hydrocarbons, air and aqueous streams

*B. C. Kibler, Antek Industrial Instruments Inc., 300 Bammel Westfield Road, Houston, TX 77090, USA*

A new analyser for the on-line analysis of total fluorides in simple and complex matrices was described. Continuous sampling combined with pyro-hydrolysis and ion specific electrode detection provide results with a dynamic range of 50 ppb to % levels. Several different sampling matrices were described, for example butanes, propane, ethylene, xylenes, air, water. Solvent usage has been minimized to reduce operating costs and to improve sensitivity. Response time of the system is measured in seconds, providing for real time measurements and control. Continuous operation and MTBF data were presented.

### *In situ* sensing of composite chemistry

*Jeffrey F. Aust, Karl S. Booksh, Christopher M. Steelman and M. L. Myrick, Department of Chemistry and Biochemistry, University of South Carolina, Columbia, SC 29208, USA*

In polymer analysis, the strength of a processed composite depends to a great extent on the chemical properties of the polymer matrix. Poor thermal conductivity coupled with internal heat generation from a cure reaction can cause temperature and reaction gradients, runaway exotherms ('hot spots'), and residual thermal stress—all of which can lead to internal damage, poor bonding between matrix and fibres, and premature structural failure. Laboratory analyses are often performed with differential scanning calorimetry (DSC), because the curing of these polymers can be controlled in a psuedo-isothermal environment. The sample in these measurements is small and thin enough that internally generated heat from exothermic polymerization is efficiently conducted from the sample. However, in industry, most polymers and composite parts are thick enough that internal heat generated by curing is not removed quickly. This makes DSC less than ideal for bulk polymer analysis. A novel probe for *in situ* fibre optic Raman spectroscopy has been tested and employed for real time monitoring of polymer curing in both thin film and bulk samples. Application of multivariate analytic techniques permit monitoring of both cure percentage and sample temperature. It is shown that spectral changes occurring during curing of thin film samples can be correlated to per cent cure as measured by differential scanning calorimetry. The probe has been employed to monitor, in real time, percent cure of polymers to within 0·35% in laboratory samples and 0·82% in industrial composities.

### Novel sensor electrode for the determination of hydrogen sulphide in environmental samples

*Ahmed Galal, Paul L. Bishop, Department of Civil and Environmental Engineering, University of Cincinnati, Cincinnati, OH 45221, USA; and Harry B. Mark, Jr., Department of Chemistry, University of Cincinnati, Cincinnati, OH 45221, USA*

Hydrogen sulphide is an important electron acceptor compound in anaerobic respiration. One important source of hydrogen sulphide ccould be the reduction of sulphate in many microbial environments. In this work the authors fabricated a hydrogen sulphide electrode made of antimony (0·25%), bismuth (44·5%), cadmium (5·3%), copper (0·10%), indium (19·1%), lead (22·45%) and tin (8·3%) alloy. The metal alloy was melted and forced down into a micropipette. For the purpose of the optimization and evaluation experiments, the tip of the micropipette was ground flush, giving a flat exposed surface of the alloy of 0·8–1·2 mm diameter. Different pretreatment methods for the alloy were evaluated, In the first treatment, the alloy was first mechanically polished using metallurgical paper of different grades, sonicated in distilled water, then subjected to a constant or stepped potentiostatic pulse in a hydrogen sulphide containing solution. For the second treatment, the alloy was polished as in the first method, a poly(3-methylthiophene) film was electrochemically formed using the cyclic voltammetric technique and then the electrode was immersed in a hydrogen sulphide solution. For the third treatment, the alloy was treated as in treatment 2, then was also subjected to a crown ether containing solution under constant potential of 2·0 V, and then subjected to the sulfide containing solution. For the fourth treatment, the alloy was polished as in treatment 1, then subjected to the crown ether containing solution as in treatment 3.

The total sulphide concentration ranges of interest are $3 \times 10^{-5}$ to $6 \times 10^{-4}$ M, and $1{\cdot}5 \times 10^{-6}$ to $3 \times 10^{-5}$ M under anaerobic and aerobic conditions, respectively. The results obtained over a much wider concentration range indicate that the electrodes prepared by each of the four methods described above provide a usable sulphide electrode. However, they were distinct with respect to: (1) the linear dynamic range, (2) stability (lifetime), (3) interferences by other ions, and (4) the pH of the solution. Sulphide electrodes prepared according to the third method showed excellent reproducibility between samples, the calibration curves obtained had a linear dynamic range in the concentration ranges expected for the sulphide ion our real samples, and had a working pH range between 6·50 and 8·00. The data were presented and discussed in terms of the considerations mentioned above.

The surfaces of the electrodes were examined using SEM, which showed differences in the morphology of the untreated and treated samples. Moreover, the structure of the surfaces were analysed using EDAX, which showed that the surface is mainly covered with the dominant constitutents of the base metals in the alloy and sulphur.

### Implementation of automated Soxhlet extraction for semivolatile analytes

*Rod Cook, Fiona Loomes, Irena Larson, Greg Pronger, NET Midwest, 3548 35th Street, Rockford, IL 61109, USA; and Kevin P. Kelly, OI Analytical, Sample Preparation Products Group, 555 Vandiver Drive, Columbia, MO 65202, USA*

Contract labs are motivated by severe competitive pressures and need methodology and equipment that reduce cost, but they are generally limited to the techniques specified in promulgated methods. Thus soils for organics analyses are usually processed by sonication or Soxhlet extraction. Automated Soxhlet Extraction (USEPA SW-846 Method 3541) is a recently accepted alternative that uses less labour than Soxhlet while providing throughputs similar to sonication extraction (six samples in parallel every two to three hours), and which minimizes solvent waste generation while providing for recovery of evaporated solvent.

Sample (30 g or 40 g) contained in a cellulose or glass thimble was suspended in beakers with 140 ml of extraction solvent (1:1 acetone and methylene chloride). The system automatically extracted the samples and evaporated the extracts to a small volume (2 to 12 ml). Previous work demonstrated that evaporation contributes negligible losses of semivolatiles in this system when evaporation is halted at 2 ml or greater volume. Thus samples are automatically extracted and evaporated in unattended fashion and the concentrate is ready for volume adjustment and clean-up or analysis.

Significant labour and solvent savings accrued for replacement of sonication extraction and Kuderna-Danish evaporation with automated Soxhlet. The authors have estimated that automated Soxhlet used 45% less solvent and eliminated 1/2 of labour costs compared to traditional Soxhlet extraction, while reducing processing time from 16 hours or more to only two hours. MDL and method precision and recovery data were presented for the SW-846 methods 8141, 8080, and 8270. For example, the system qualified for 8141 with MDLs ranging from 0·39 to 1·8 µg/kg. Average recoveries for 10 spiked triazines (4 replicates at 33 or 100 µg/kg) were 95% to 119% (except metribuzin at 17 µg/kg, 84%) and the RSDs ranged from 2·4% to 9·2%. Also presented were the comparative recovery results for soils extracted using the automated Soxhlet and traditional extraction methods.

### Real time automation of environmental GC/MS analysis

*Charles R. Lyle, Steven D. Cubbege, William R. Dougherty, Shimadzu Scientific Instruments Inc. 7102 Riverwood Dr., Colombia, MD 21046, USA; N. Mukai and I. Kosaka, Shimadzu Corporation, 1 Nishinokyo-Kuwabaracho, Nakagyo-ku, Kyoto, 604 Japan*

The automation of analysis by GC/MS has improved steadily over the years to the point where it is now possible to automate the performance of Tune Criteria Checking for EPA Methodology. The operator is the most expensive and time consuming part in the analysis of environmental samples. The Environmental Protection Agency specifies the interval and ion fragment acceptance criteria for each analytical method this value can be as little as eight hours and as long as 12 hours. Minimizing the requirement of operator intervention into sample processing to perform this function along with reducing the sample analysis time are the most productive ways to improve instrument throughput in the environmental laboratory.

Improving the productivity of the laboratory is the goal of the Shimadzu Corporation. The uninterrupted processing of samples for the longest possible time and the shortening of the time necessary for each analysis improves each instrument's productivity and also that of the entire laboratory. The Shimadzu QP-5000 system incorporates advanced, exceptionally stable, hardware with software that automatically aquires the tuning compound, creates background subtracted spectra and performs a check of acceptance criteria check with no operator intervention. If the criteria are met, the system continues with the sample schedule and prints a report showing that the performance is within its programmed limits. When there is a possible matrix concern, the system can be programmed to perform an automatic tune before the tuning check sample is injected.

Sample analysis time is the other area where dramatic improvements have been made in improving instrument productivity. By combining the latest in narrow-bore capillary technology with the GC17 Gas Chromatograph, the QP-5000 system can analyse samples faster than the cycle time of the purge and trap concentrator. Data were provided demonstrating the tune stability, initial calibrations, continuing calibration and surrogate recovery for these improved analytical techniques.

### Application of multicomponent analysis and sequential injection analysis for the rapid determination of total nitrogen

*M. T. Oms, V. Cerdà, and A. Cerdà, Department of Chemistry, University of the Balearic Islands 07071-Palma de Mallorca, Spain*

An automatic method for the determination of total nitrogen in wastewater using sequential injection analysis and mineralization with UV radiation was developed. The method is based on the mineralization of the samples with sodium persulphate in alkaline medium under UV radiation. Small volumes of the sample and reagents are firstly aspirated into a single channel and then propelled by flow reversal to the UV reactor where the organic and inorganic nitrogen compounds are oxidized to nitrate. The sample is then propelled to the detector and the spectrum in the range 200–320 nm is obtained every second. Multicomponent analysis is applied to the spectra obtained at the peak maximum to discriminate between the signal due to nitrate ions and that due to the matrix and excess of persulphate.

The sequential injection procedure has been optimized and the factors affecting the efficiency of the oxidation have been studied using test substances with different chemical structures and properties. Solutions in the concentration range 0–60 mg $l^{-1}$ of nitrogen can be analysed with the described procedure. The LOD is 0·6 mg $l^{-1}$ N and the reproducibility is 1·8% (28 mg $l^{-1}$ N). Organic carbon in the form of glucose was added to the test solutions to study the potential interference of organic matter. For samples with high content of organic matter, for example raw wastewater samples, the determination is linear up to 25 mg $l^{-1}$ N.

The performance of this method for wastewater analysis was compared with the traditional Kjeldahl digestion. The nitrate and nitrite content of the unoxidized samples were substrated from the corresponding nitrogen content determined after photo-oxidation and the value compared with the Kjeldahl nitrogen content.

### Determination of arsenic in food, biological, and environmental samples at ultra-trace levels using vapour generation atomic fluorescence spectrometry

*R. Fernando, M. Goldberg, E. D. Pellizzari, L. S. Sheldon and M. Lang, Research Triangle Institute, Dreyfus Building, PO Box 12194, RTP, NC 27709, USA*

Methods were developed to determine arsenic at ultra-trace levels in a wide variety of sample matrices including composite food, biological tissues, aerosol, soil, and house dust using vapour generation atomic fluorescence spectrometry.

Sample preparation methods for composite food and biological tissues involved homogenization of the sample, digestion with nitric acid and hydrogen peroxide at 85°C, followed by open vessel volume reduction in a microwave. Preparation of aerosol, soil, and house dust involved extraction of samples with 50% (v/v) nitric acid at room temperature, followed by open vessel volume reduction on a hot plate.

Sample analysis involved pre-reduction of As(V) to As(III) by the addition of potassium iodide and ascorbic acid reagent and formation of arsine ($AsH_3$) with the addition of sodium tetrahydroborate. Atomic fluorescence intensity was measured using a P. S. Analytical (Orpington, UK) atomic fluorescence spectrometer.

The performance of the digestion and the analysis methods were evaluated by using appropriate standard reference materials (SRMs), NIST certified samples with low levels of arsenic, and spiked samples. Analytical methods have resulted in method detection limits, calculated as three times the standard deviation of replicate method blanks, in the range 6 to 9 pg of As/ml. Method performance evaluation results and sample analysis results were presented.

### Evaluation of method 525·2 using automated solid phase extraction

*Mark Krigbaum, Tekmar Company, PO Box 429576, 7143 East Kemper Road, Cincinnati, OH 45242-9576, USA*

Historically, semi-volatiles in drinking water have been extracted by separatory funnels and liquid-liquid continuous extractors. These techniques require large volumes of solvent which are expensive to purchase and dispose of. Solvents also affect laboratory personnel safety and environmental safety.

Solid Phase Extraction (SPE) is a technique that environmental laboratories use to reduce the time and amount of solvent consumed. Manual SPE followed by a concentration step can be labour intensive, time consuming and prone to error. This work showed that, by automating SPE, the goal of speed and high quality data can be met.

This study focused on USEPA method 525·2. Analytes were extracted from drinking water by an automated SPE workstation and recoveries were determined by gas chromatography mass spectrometry. Analyte recoveries, method detection limits, and repeatability were evaluated.

### The on-line determination of total olefins in gasoline by process gas chromatography

*Robert K. Bade, Stephen B. Hunt, Gerald L. Combs and Eugene L. Kesselhuth, Applied Automation Inc., PO Box 9999, Bartlesville, OK 74006-9999, USA*

Total olefins in gasoline can now be determined by On-Line Process Gas Chromatography (PGC). A method has been developed that allows the determination to be done rapidly and very repeatably. The cycle time (injection to injection) is 20 minutes and the relative standard deviation is 0·35% at 12% olefins. The technique uses a packed column (OV-275) to remove the aromatic compounds. The non-aromatics are transferred in two groups onto a silver ion exchange column, which subsequently separates the non-olefins from the olefins in each group. After elution of the non-olefins the column is rapidly heated and flow reversed to elute the olefins as a group. The unique column switching arrangement involves both valve and pressure switching. A flame ionization detector is used. The paper detailed interferences and compared results with laboratory FIA results. A correlation coefficient of 0·97 has been obtained. A commercial instrument, the Applied Automation Advance Gas Chromatograph, was described which allows implementation of this method in a Division 1 Group C & D hazardous area.

